# German consumers’ attitudes toward artificial meat

**DOI:** 10.3389/fnut.2024.1401715

**Published:** 2024-06-12

**Authors:** Anne-Katrin Jacobs, Hans-Wilhelm Windhorst, Julia Gickel, Sghaier Chriki, Jean-François Hocquette, Marie-Pierre Ellies-Oury

**Affiliations:** ^1^Science and Innovation for Sustainable Poultry Production (WING), University of Veterinary Medicine Hannover (TiHo), Foundation, Field Station for Epidemiology (Bakum), Bakum, Germany; ^2^Department II - Natural and Social Sciences, University of Vechta, Vechta, Germany; ^3^Université Clermont Auvergne, Institut National de Recherche pour l’Agriculture, l’Alimentation et l’Environnement (INRAE), VetAgroSup, UMR1213, Recherches sur les Herbivores, Theix, Saint-Genès-Champanelle, France; ^4^Isara, Lyon, France; ^5^Bordeaux Sciences Agro, Bordeaux, France

**Keywords:** cultured meat, consumer perception, traditional agriculture, ethics, novel food

## Abstract

The aim of this study was to analyze the impact of sociodemographic characteristics on willingness to try (WTT), regularly eat (WTE), or pay (WTP) for artificial meat, its expected societal challenges and general acceptance as well as its future potential development in Germany. Answers to an online questionnaire by 3,558 potential German adult consumers were evaluated. About 63% of the respondents thought this novel food was promising/acceptable. The vast majority (70%) stated that they would be willing to try it, with the most important drivers being ethics, curiosity and eco-friendliness. Around 57% of the participants said they would be willing to eat artificial meat regularly. Most of the respondents (40%) were willing to pay the same price for artificial as for conventional meat. In terms of its future potential, almost 75% of respondents believed that this new product would become commercialized in more than five years and that it was perceived as a solution that is both more ethical (67%) and more environmentally friendly (58%) than traditional meat. In addition, there were significant impacts of demographic factors on the willingness to engage with artificial meat. For example, high WTT and WTE were found among young male respondents (18–30 years of age), males that rarely consumed meat or had a low income (< €1,500). This also applied to the female respondents, who, however, belonged to higher income classes. Young German consumers with a high level of education or income up to €3,000 as well as consumers who did not eat meat had a high WTP for this novel food. In addition, respondents’ positive opinion and acceptance of artificial meat had a positive influence on WTT and WTP. These results are important for the discussion of a paradigm shift in global meat production with respect to sustainability, demand for meat and the adoption of new food products.

## 1 Introduction

According to a United Nations’ (UN) projection, the global population will increase from 8.5 billion in 2030 to 9.7 billion in 2050 (1), and early projections estimated that around 70% more meat in 2050 will be needed to meet future demand (2). However, a recent study found that future meat demand varies depending on the type of meat. In the period from 2019 to 2050, a global increase in demand for beef of 19%, for pork of 39% and for poultry of 131% was projected. Meat as a whole, together with the rising demand for fish, was calculated with a total global increase of 67% (3). Sijpestijn et al. (4) noticed that “the current model of agriculture has the capacity to meet global protein requirements,” but “it fails to do so through lack of access, inefficiencies and losses.” In order to tighten the expected widening gap between production and demand, new food systems have been developed. These alternative systems can either be new farming systems based on agroecology (5), use of insects, plant based products, or even use cell culture technologies or fermentation (6). Artificial meat (AM), or “cell-based food” as recommended by the FAO (7), belongs to the novel foods. It is grown in a nutrient solution using animal muscle stem cells, the cells come from alive animals by biopsies. AM production is the subject of media hype, which is mainly related to an increase in demand for food due to a growing population and to new social expectations regarding environmental issues and animal welfare concerns. So far, only four artificial meat products have been approved for the market: in Singapore (Eat Just) (8), in the USA (Upside Foods and Good Meat (9)) and in Israel (Aleph Farms) (10).

Over the past decade, overall *per capita* meat consumption in Germany has decreased, even by 8% from 2021 to 2022 (11) whereas sales of plant-based meat in particular increasing by more than 42% in 2022 compared to 2020 (12). In addition, the proportion of vegetarians and vegans increased from 6% in 2020 to 10% in 2023 (13). Consumers like flexitarians explained their reduction in meat consumption in particular by ethical, ecological and health motives (14, 15). Nevertheless, traditional meat is part of the diet for most Germans. Our survey will document attitudes of potential German consumers toward AM. To specify this, we have made the following hypotheses (H), which should either be verified or refuted by a survey of potential German consumers:

*H1a/1b*: (a) Due to the continuously decreasing consumption of conventional meat in Germany and the increasing demand for meat alternatives, there may be a great interest in trying and eating artificial meat mainly due to positive opinions about artificial meat. (b) Respondents’ positive opinions about artificial meat will have a very positive impact on trying and eating this new product.

*H2*: Demographic and economic factors are likely to play a major role in the adoption of this new product. For instance, young and well-educated German consumers may be more open to AM.

*H3*: It is also well known that consumers pay particular attention to price when buying food. With regard to AM, respondents may not be willing to pay a higher price than for conventional meat.

*H4*: Ethical, ecological and health concerns are the main reasons for reducing meat consumption in Germany. Potential German consumers will also perceive artificial meat as a solution to ethical and ecological problems of conventional meat production.

*H5*: As the sale of the novel food was only approved in Singapore at the time of the survey, the respondents may not expect it to be implemented in Germany in the very near future despite huge investments and advertising in the public media.

This survey was part of a much broader international study on AM initiated by French scientists from INRAE, ISARA and Bordeaux Sciences Agro. Previous results were published independently for Africa (16), Brazil (17), China (18), France (19) and Spain, Italy and Portugal (20). The results of this German study were not part of the previous analyses in previous publications.

Nevertheless, in order to ensure the comparability of the data from other countries in the international research project, the most important variables were included in the German analysis based on the previous results for Africa, Brazil, China, France as well as Spain, Italy and Portugal.

## 2 Materials and methods

### 2.1 Questionnaire design

The German evaluation was part of an international project. Before the survey also took place in Germany, the questionnaire was validated and performed in several other countries (France, Brazil, China, Italy, Portugal, Spain, etc.) with thousands of respondents and always led to significant results.

The questionnaire was first designed in English to be distributed in different English speaking countries (UK, US, Australia, NZ) and translated into different languages (French, German, Chinese, Portuguese, Italian, Spain and Arabic) to be distributed in the corresponding countries. For this reason, the design of the questionnaire for the German survey (Supplementary Figure A1) was completely assumed from the original English version.

A total of 33 questions were translated from English into German by native German speakers. The questionnaire was formatted in LimeSurvey (21), which is a free online application.

First of all, we described the background of the survey and ensured compliance with the Code of Ethics of the survey institutions, legal data protection requirements and anonymity of the survey without any personally identifiable information. It should be noted that the survey complied fully with the Lower Saxony Data Protection Act (NDSG) (22). For this purpose, a data security declaration with information on data protection and a declaration of consent (purpose of processing, legal basis for processing, duration of processing, rights of data subjects) have been drawn up. The use of the data for teaching, research, lecture and publication purposes is based on Art. 6 I 1 lit. and i. V. m. § 13 NDSG (22).

Then, the principles of producing AM were explained by means of a figure. The respondents gave their explicit consent to take part in the survey and assured that they were informed about the protection of their personal data. The questions (including consent to the transfer of data) were divided into seven main parts:

(1) Demographic information:

- Continent or country of origin, gender, age (“31–50 years of age,” “31–50 years of age,” “51 years of age and more”; closed-ended single choice question),
- Education (“Secondary modern school,” “University-entrance diploma,” “University degree,” “PhD,” “Do not want to answer,” “Others”; half-open single choice question), the answers were assumed to match the German school system,
- Area of work (“Scientist within the meat sector,” “Scientist outside the meat sector,” “Not scientist but within the meat sector,” “Not scientist and outside the meat sector”; closed-ended single choice question),
- Monthly net income (“< €1,500,” “€1,500 – €2,000,” “€2,000 – €2,500,” “€2,500 – €3,000,” “€3,000 – €4,000,” “> €4,000,” “Does not wish to answer”; closed-ended single choice question),
- Level of meat consumption (“Never: vegetarian or vegan diet,” “Rarely: weekly or less,” “Regularly: several times a week,” “Daily or within each meal”; closed-ended single choice question),
- Familiarity with artificial meat (“Yes, “No”) (closed-ended single choice question).

(2) Preamble with two introductory questions: “Have you ever heard of artificial meat?” (“Yes,” “No”) and “What are the most important criteria when you do your food shopping?” (half-open multiple choice question).

(3) Societal challenges: Perception of the challenges (ethical and environmental issues) facing the livestock industry and meat production as well as the opinion on reducing meat consumption (7 questions, 5-point Likert scale ranging from disagreement to agreement).

(4) Characteristics of the product: Assuming how healthy, safe, nutritious and tasty artificial meat will be compared to conventional meat (2 questions, 5-point Likert scale ranging from “much less” to “much more”).

(5) Potential interests:

- Artificial meat as a viable alternative compared to conventional meat (closed-ended single choice question),
- Reasons for WTT or not WTT (half-open multiple choice questions) and
- Expectations about artificial meat (half-open multiple choice questions).

(6) Perceptions about artificial meat:

- “What do you think of artificial meat?” (“It is promising and/or acceptable,” “It is fun and/or intriguing” or “It is absurd and/or disgusting”; closed-ended single choice question),
- Emotional resistance to try artificial meat (5-point Likert scale ranging from “much less” to “much more”),
- WTT (“Definitely yes,” “Probably yes,” “Unsure,” “Probably not,” “Definitely not”; closed-ended single choice question),
- WTE (“At the restaurant,” “At home,” “In ready-to-eat meals,” “I do not want to eat artificial meat regularly,” “Other”; half-open single choice question),
- WTP (“Much less than conventional meat, even nothing at all,” “Less than conventional meat,” “Same price as conventional meat,” “More than conventional meat,” “Much more than conventional meat”; closed-ended single choice question).

(7) Development strategies:

- Opinion when artificial meat becomes realistic (“On the short term: from 1 to 5 years,” “On the medium term: from 6 to 15 years,” “On the long term: more than 16 years,” “Never”; closed-ended single choice question).
- Future naming (half-open multiple choice question).
- Should it be named “meat” when commercialized one day (“Yes,” “No”; closed-ended single choice question).
- The relevance of private and public research projects for development strategies (2 questions, 5-point Likert scale ranging from “Much less” to “Much more”).

### 2.2 Data collection

To ensure that the German questionnaire was formulated in a comprehensible way, a pretest was made with 10 native German speaking people from the scientific and the non-scientific sectors. Afterwards, an email, containing an explanatory text and the link to the final web survey “Umfrage zu künstlichem Fleisch” (“survey on artificial meat”), was sent to various people (in non-governmental organizations, in educational and research institutions, in the public media, in companies along the food value chain and to politics) inviting them to take part in the survey. Additionally, the survey was advertised in various groups and pages on Facebook during the survey period (i.e., the homepage of the University of Veterinary Medicine Hannover). Fifteen minutes were estimated for completing the questionnaire.

The German web survey was online in Germany from January 15, 2021 to January 25, 2022 because the authors aimed to obtain more than 4,000 fully completed questionnaires for Germany. The answers were administered by using LimeSurvey (21). All respondents with more than 18 years of age were defined as the target group.

Overall, a total of 4,713 questionnaires were collected. Of these, 4,036 were fully completed, but only 3,875 of the respondents agreed to save their data at the end of the questionnaire. In order to analyze the behavior of the consumers that are familiar with the German market, only those who answered German for “origin” were included in the evaluation (17). The survey thus comprises 3,558 responses, which were further analyzed.

### 2.3 Statistical analysis

The data were analyzed using IBM SPSS statistics (version 27) (23), R studio (version 2022.07.1554.0 and a previous version) (24) as well as Microsoft Excel 2016 (25). The statistical part was based on the analyses conducted in other publications of the project (17, 18).

The demographic variables were described with SPSS “descriptive statistics,” based on the number and percentage of responses (3,558 = 100%), mean and standard deviation (SD). Then the responses concerning “societal challenges related to the meat industry,” “perceptions about artificial meat” and the “emotional resistance for willingness to try artificial meat (WTT)” were scaled using the 5-point Likert scale from “much less” (scale 1) to “much more” (scale 5) and described also based on the number and percentage of responses, mean and SD with SPSS “descriptive statistics.” To examine perceptions about AM regarding trying (WTT), price (willingness to pay in relation to conventional meat; WTP) and consumption (willingness to eat artificial meat regularly; WTE), the variables were coded as follow:

- for WTT: from “Definitely yes” = 5 to “Definitely not” = 1,
- for WTP: from “Much less than conventional meat, even nothing at all” = 1 to “Much more than conventional meat” = 5 (5-point Likert scale),
- for WTE: “Not willing to eat artificial meat regularly” = 0 and “Willing to eat artificial meat regularly” = 1 (a summary of the responses: at the restaurant, at home, in ready-to-eat meals and others).

The following step in the analysis was to examine the effects of demographic factors on the willingness variables (WTT, WTE and WTP). As in the Chinese part of the project, for example, the assumptions of the ANOVA were partially violated, such as the homogeneity of the variances or the normality of the distributions (18). The variance of WTT, WTE and WTP according to demographics was calculated using Welch’s ANOVA, which does not require an assumption of homogeneity of variance. To correct for multiple testing, a Tukey-B adjustment was used. As in previous analysis (18), the results were very similar compared to ANOVA. For the pairwise comparisons between significant demographic groups and the willingness variables, a two-factor analysis of variance was required. As Welch’s ANOVA does not accept interactions, the ANOVA was chosen and completed with a Bonferroni adjustment. Only significant interactions were indicated. Significance tests were considered at a level of *p* < 0.05. In addition, a mosaic plot was computed for the variable WTP in relation to the pair of variables with the highest significance (Gender x Income, *p* = 0.000061). The mosaic plot was completed using the “mosaic” and “vcd” packages in R.

For the analysis of the driving factors of willingness to engage with AM, the positive and negative correlations for “societal challenges” (Does farm animal husbandry/meat industry cause ethical problems?; Does farm animal husbandry/meat industry cause environmental problems?; Could reducing our meat consumption be a solution?; How would you compare artificial meat ethically to conventional meat?; How would you compare artificial meat from an environmentally friendly point of view to conventional meat?; Will artificial meat have a negative impact on conventional livestock farming and the meat industry?; Will artificial meat have a negative impact on rural areas and rural life?) and WTT, WTE and WTP were highlighted. As it is usual for complex datasets, exploratory techniques were used. First of all, non-parametric correlations were analyzed (with SPSS “correlation”) and were performed using a correlation matrix plot in Excel. All correlations were marked as significant with Spearman-Rho at the 0.01 level (two-sided). In addition, a Principal Component Analysis (PCA) was conducted in R with several libraries (“carData,” “car” and “ellipse”). The goal of the PCA was to summarize the variables of the “social challenges” part as well as WTT, WTE and WTP into a few dimensions and to extract the principle components (PCs). The ellipses for the qualitative variables “Acceptance” (Would you accept artificial meat as a viable alternative compared to conventional meat in the future…?), “Opinion” (What do you think of artificial meat?), “Commercialization” (Do you think artificial meat is realistic?) and “Name” (If this product is commercialized one day, do you think it should be named “meat”?) were mapped. The PCs with variances greater than 1, in this case the dimensions PC1 and PC2, were considered (26).

In a final step, cross-analyses were computed in SPSS for analyzing the relationships among respondents´ attitudes, perspective and acceptance of WTT and WTE. In a final step, three cross-analyses were computed in SPSS for analyzing the relationships among respondents´ attitudes, perspective and acceptance of WTT and WTE:

1. Attitude: WTT versus WTE in different settings (at home, at the restaurant, in ready-to-eat meals) (in %; multiple choice),
2. Acceptance of AM as an alternative to conventional meat versus WTE/WTT (in %),
3. Perspective of AM (promising/acceptable, fun/intriguing, absurd/disgusting) versus WTE in different settings (at home, at the restaurant, in ready-to-eat meals) (in %; multiple choice).

## 3 Results

### 3.1 Population characteristics

First of all, it should be noted that our sample did not correspond in all respects to the socio-demographic structure of the adult German population. A comparison can be found in section 4.6 “Limits and strengths of the survey.”

Among the 3,558 persons included in this survey, 59.8% were women and 38.3% were men (1.9% of respondents chose not to state their gender) (Table 1). On average, respondents were evenly distributed between the two age categories: 18–30 (38%) and 31–50 years of age (41.5%). People over the age of 50 accounted for 20.5%. Almost 60% of respondents were well educated consumers (medium or high level of education) with medium or low incomes (< €2,500; 54.7%). Most of the respondents worked outside the meat sector (89.3%). Nearly 95% of respondents had already heard of AM and therefore seem to have been well informed. A total of 70.1% of the respondents were meat eaters but with different frequencies. German respondents’ opinions of the new biotechnology in this study were predominantly “promising/acceptable” (63%), followed by “absurd/disgusting” (22.4%) and “fun/intriguing” (14%).

**Table 1:** Demographic information on the survey respondents.

Attribute	Response option	*N*	% (3,558 = 100%)
Gender	Female	2,128	59.8
	Male	1,364	38.3
	Does not wish to answer	66	1.9
Age	18–30 years of age	1,352	38.0
	31–50 years of age	1,477	41.5
	≥ 51 years of age	729	20.5
Education	Secondary modern school	428	12.0
	University-entrance diploma	940	26.4
	University degree	1,668	46.9
	PhD	448	12.6
	Does not wish to answer	44	1.2
	Others (e.g. Master’s, student)	30	0.8
Area of work	Scientist: meat sector	95	2.7
	Scientist: other sector	643	18.1
	Worker: meat sector	288	8.1
	Worker: other sector	2,532	71.2
Monthly net income	< €1,500	954	26.8
	€1,500 – €2,000	459	12.9
	€2,000 – €2,500	535	15.0
	€2,500 – €3,000	413	11.6
	€3,000 – €4,000	407	11.4
	> €4,000	374	10.5
	Does not wish to answer	416	11.7
Meat consumption	Never: vegetarian or vegan diet	1,064	29.9
	Rarely: weekly or less	926	26.0
	Regularly: several times a week	1,343	37.7
	Daily or at each meal	225	6.3
Familiarity with artificial meat	Yes	3,371	94.7
	No	187	5.3

### 3.2 Perceptions of societal challenges related to the meat industry

The majority of respondents believed that the traditional meat industry was currently facing ethical (score 5: 58.2%) and environmental (score 5: 59.6%) problems and that reducing meat consumption was likely to be a solution to some of these problems (score 5: 58.0%) (Supplementary Table A1). Most respondents believed that AM was both a more ethical (scores 4 and 5: 66.7%) and environmentally friendly (scores 4 and 5: 57.7%) solution than traditional meat. Over half of the respondents agreed that this technology would have negative impacts on the meat industry (scores 4 and 5: 54.2%). Nearly half of the respondents considered that AM would have equivalent properties in terms of sanitary, nutritional and sensory quality (score 3: 48.1%). A lower proportion of respondents was convinced that AM would taste better than conventional meat (scores 4 and 5: 15.2%) and there was even a higher percentage who thought it would not taste as good (“less,” scores 1 and 2: 32.1%). Slightly more than half of the respondents expressed a neutral opinion (score 3: 52.6%). Compared to 29.6% of the respondents who indicated that they had high emotional resistance to WTT (scores 4 and 5), 53.1% had less emotional resistance (scores 1 and 2), whereas 17.3% of them were unsure (score 3).

### 3.3 Engagement with AM and its future development

#### 3.3.1 Willingness to engage and development strategies

Nearly 70% of the respondents would be willing to try AM (definitely or probably) with only 23.3% (definitely or probably) would refuse to try it (Table 2). Especially at home, respondents could imagine regularly consuming AM (46.5%), and in similar proportions in restaurants and ready-to-eat meals (36.6%). Nevertheless, 42.8% did not wish to eat this new product regularly.

**Table 2:** Responses on willingness to engage in artificial meat and its future development.

Questions: Willingness to try, to eat regularly and to pay for artificial meat	No.	%	Mean	SD
Would you be willing to try artificial meat?				
1. Definitely yes	1,485	41.7		
2. Probably yes	965	27.1		
3. Unsure	279	7.8	2.24	1.39
4. Probably not	418	11.7		
5. Definitely not	411	11.6		
Would you accept artificial meat as an alternative to conventional meat compared to meat substitutes?				
1. Yes, and I eat meat substitutes	1,623	45.6		
2. Yes, but I do not eat meat substitutes	742	20.9	2.09	1.19
3. No, but I eat meat substitutes	432	12.1		
4. No, and I do not eat meat substitutes	761	21.4		
In which context(s) would you be willing to eat artificial meat regularly? (multiple choice)				
At the restaurant	1,303	36.6		
At home	1,653	46.5	-	-
In ready-to-eat meals: lasagna, burgers…	1,304	36.6		
I do not want to eat artificial meat regularly	1,523	42.8		
How much would you be willing to pay for artificial meat compared to conventional meat?				
1. Much less than for conventional meat, even nothing	542	15.2		
2. Less than for conventional meat	623	17.5		
3. Same price as for conventional meat	1,419	39.9	2.82	1.05
4. More than for conventional meat	873	24.5		
5. Much more than for conventional meat	101	2.8		
Questions: Development and future	No.	%	Mean	SD
When do you think artificial meat will become realistic?				
1. Short-term: 1 to 5 years	218	6.1		
2. Medium-term: 6 to 15 years	1,517	42.6		
3. Long-term: >16 years	1,139	32.0	2.64	0.85
4. Never	684	19.2		
Do you think a private research model (start-ups) is relevant for potentially developing research?				
1. Much less	157	4.4		
2. Less	178	5.0		
3. Neutral	963	27.1	3.74	1.02
4. More	1,388	39.0		
5. Much more	872	24.5		
Do you think that public research must invest (time and funding) to develop this biotechnology?				
1. Much less	412	11.6		
2. Less	292	8.2		
3. Neutral	666	18.7	3.61	1.31
4. More	1,080	30.4		
5. Much more	1,108	31.1		

Overall, 57.7% of the respondents were consumers of meat alternatives. Among them, 45.6% would accept AM as an alternative to conventional meat compared to meat substitutes, while 12.1% would deny this. In total, 42.3% of the respondents did not eat meat alternatives at all. However, 20.9% of them would choose this new product as a meat substitute.

Overall, regarding WTP, 32.7% said AM would be acceptable if sold at a lower price than traditional meat. A majority of 39.9% would like to pay the same price as for conventional meat.

Nearly 75% believed that AM would be realistic in the medium and long term (i.e., “in 6 to 15 years” and “in more than 16 years”) (Table 2). Only 6% of respondents thought that AM would be implemented within one to five years. In total, 19.2% of respondents thought that this technology would never take off.

Furthermore, our German respondents thought that a private research model was relevant for future research in AM (63.5%, i.e., “more” and “much more”). Similarly, 61.5% (i.e., “more” and “much more”) believed that it was the task of public research to develop this new technology with time and financial resources.

#### 3.3.2 Reasons for engaging, obstacles, and expectations of AM

**Figure 1:**
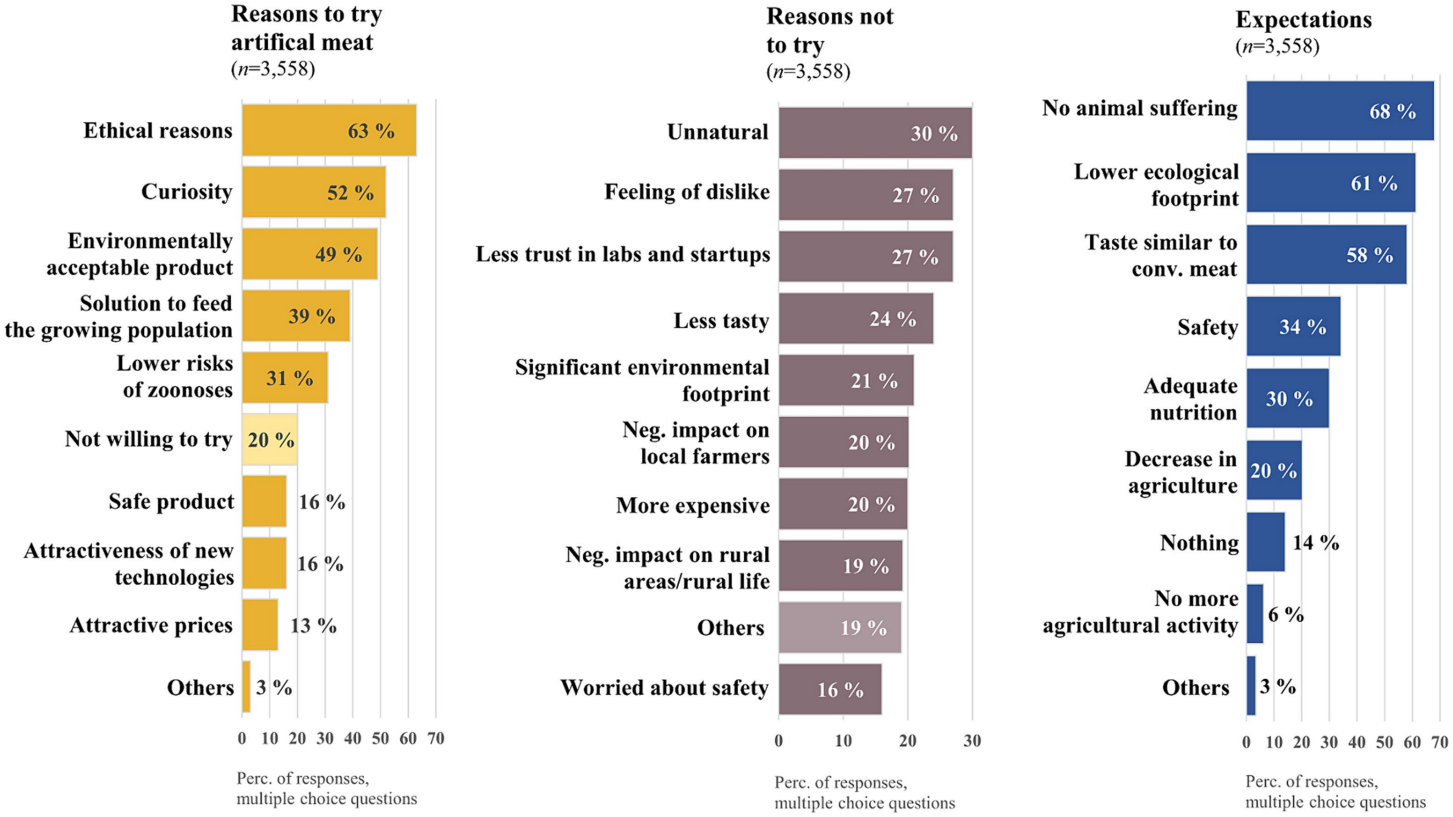
Reasons for engagement, obstacles and expectations for artificial meat (multiple choice questions).

Among the most important drivers for the German respondents regarding WTT were (multiple choice): ethics (63%), curiosity (52%) and eco-friendliness (49%) (Figure 1). A total of 20% of respondents were not comfortable with the idea of WTT.

Unnaturalness (30%), a feeling of dislike (27%), less trust in labs and startups in the new sector (27%), and less taste (24%) have been reported as obstacles to WTT (multiple choice). Only 16% of the respondents were concerned about the safety of this new product. The vast majority of the participants supported the statement that AM would be free of animal suffering (68%), would have a smaller environmental footprint (61%) and would taste similar to conventional meat (58%). The respondents suspected that there would be a decrease in conventional agriculture due to AM (20%). Only 6% of respondents assumed a total future abandonment of previous agricultural activities through the implementation of this new product.

### 3.4 Effects of demographic factors on willingness to try, to eat regularly or to pay for AM

The following analyses are divided into two sections. In a first step, WTT is examined with regard to the correlations with the demographic factors (gender, age, education, income, meat consumption and awareness of the new product). In a next step, the interactions between demographic factors regarding WTE and WTP are surveyed.

#### 3.4.1 Willingness to try (WTT)

Each of the seven studied demographic factors was significant (*p* < 0.05) in terms of effects on WTT (Table 3). Table 3 also includes the significant interactions between these factors.

**Table 3:** Analysis of variance of willingness to try as a function of demographics.

Willingness to try			
Demographic factors	*p* value	Significant interactions	*p* value
Sex	< 0.001	Sex x Age	0.002
Age	< 0.001	Sex x Income	< 0.001
Education	< 0.001	Sex x Meat consumption	< 0.001
Area of work	0.002	Age x Education	0.004
Income	< 0.001	Age x Meat consumption	0.001
Meat consumption	< 0.001	Education x Income	0.041
Familiarity	< 0.001	Area of Work x Income	0.011
		Income x Meat consumption	< 0.001

The mean difference is significant at the 0.05 level. Only significant interactions are indicated.

With increasing age, WTT of respondents decreased regardless of indicated gender (Supplementary Table A2). It is striking that male respondents with low incomes (< €1,500) showed the highest WTT (4.20). Females had the highest WTT in the both income classes €1,500 – €2,000 (3.91) and €3,000 – €4,000 (3.94). In particular, female and male respondents who rarely (weekly or less frequently) consumed meat had a higher WTT (4.17 and 4.41, respectively) than those who included meat in their daily diet (3.20 and 3.31, respectively). Female vegetarians or vegans had a lower WTT (3.48) compared to men with this eating habit (3.84). In addition, in each of the three age groups, those who rarely consumed meat had a higher WTT than those with a daily meat consumption. Respondents over 50 years of age who ate meat regularly had the lowest WTT (3.23). Furthermore, based on age, young respondents (18–30-year-old) with a PhD degree had the highest WTT (4.34). However, those in this age group (18–30-year-old) who had a school leaving qualification had the least WTT (3.16).

Comparing the different education groups with the six income levels, those with a PhD and an income between €2,000 and €2,500 had the highest WTT (4.44).

While workers in the meat sector with the lowest incomes (up to €2,000) also had the lowest WTT (3.05 and 2.81, respectively), scientists from the meat sector with a median income between €2,000 and €2,500 had the highest WTT (4.45).

Finally, the results revealed that those with incomes between €2,000 and €2,500 and those with high incomes of more than €4,000 with a rare meat consumption had the highest WTT (4.34 each).

#### 3.4.2 Willingness to eat regularly (WTE) and to pay (WTP) for AM

The demographic factors were significant at the 0.05 level for the analysis of variance of WTE and WTP (Table 4). The significant interactions between the variables for these research questions were also shown.

**Table 4:** Analysis of variance of willingness to eat regularly and of willingness to pay as a function of demographics.

Willingness to eat regularly			
Demographic factors	*p* value	Significant interactions	*p* value
Sex	0.009	Sex x Age	0.007
Age	< 0.001	Sex x Meat consumption	0.000
Education	< 0.001	Age x Income	0.026
Area of work	< 0.001	Age x Meat consumption	0.005
Income	< 0.001	Income x Meat consumption	0.000
Meat consumption	< 0.001		
Familiarity	0.041		
Willingness to eat regularly			
Demographic factors	*p* value	Significant interactions	*p* value
Sex	< 0.0001	Sex x Age	< 0.001
Age	< 0.0001	Sex x Income	< 0.0001
Education	< 0.0001	Sex x Meat consumption	< 0.0001
Area of work	0.026	Age x Education	0.004
Income	< 0.0001	Age x Income	0.013
Meat consumption	< 0.0001	Age x Meat consumption	0.002
Familiarity	< 0.0001	Education x Income	0.025
		Income x Meat consumption	0.031

The mean difference is significant at the 0.05 level. Only significant interactions are indicated.

The results showed that the youngest female and male respondents between 18 and 30 years of age had the highest WTE (0.59 and 0.69, respectively) (Supplementary Table A3). Male respondents with the lowest income had the highest WTE (0.70) whereas females had the highest WTE in both income classes €2,000 – €2,500 and €3,000 – €4,000 (both 0.57). However, male and female respondents who rated their meat consumption as “rare” had the highest mean score for WTE (0.72 and 0.65, respectively). Male participants who reported never eating meat had higher WTE than female vegetarians or vegans.

Respondents aged between 18 and 30 years in the four income classes up to €3,000 had the highest WTE (0.64, 0.61, 0.60, and 0.63, respectively). In contrast, respondents aged over 50 years in the lowest income class had the lowest WTE (0.26). While the 18–30-year-old respondents who ate meat rarely had the highest WTE (0.76), the lowest WTE was seen in those over 50 years of age who regularly ate meat (0.32).

The analyses further indicated that respondents in the income class < €1,500 with a rarely or daily consumption of traditional meat products had the highest mean value for WTE (0.74 and 0.68, respectively). At the same time, the lowest WTE was observed for respondents in the income group €2,500 – €3,000 with a daily consumption of meat (0.19).

The next step was to analyze pairwise comparisons between demographic groups significant for WTP. Younger females and males (18–30 years of age) had the highest WTP (3.09 and 2.94, respectively) whereas older males (≥ 51 years of age) had the lowest mean value for WTP (2.29) (Supplementary Table A4). In terms of income, males had a lower WTP than females in the respective classes. Both female and male vegetarians and vegans had the highest WTP compared to those who ate meat, regardless of frequency.

With regard to the age groups, 18–30-year-old consumers with a university entrance qualification, university degree and PhD had the highest mean WTP value (3.11, 3.06 and 3.07, respectively). In addition, this age group had the highest WTP when their income reached €3,000. Among all age groups, those who did not eat meat had the highest WTP. In contrast, WTP was the lowest among 31–50-year-old consumers who reported daily meat consumption (2.02).

Those with a school leaving qualification and an income above €4,000 had the lowest WTP (2.04). On the other hand, well-educated people with a PhD and an income between €2,000 and €2,500 had the highest WTP (3.12). With exception of the €2,500 – €3,000 category, German adults with a school leaving qualification had a lower WTP in all income classes than those with other education. The comparison between income and meat consumption shows that in all income classes vegetarians and vegans had a higher WTP than the meat eaters.

**Figure 2:**
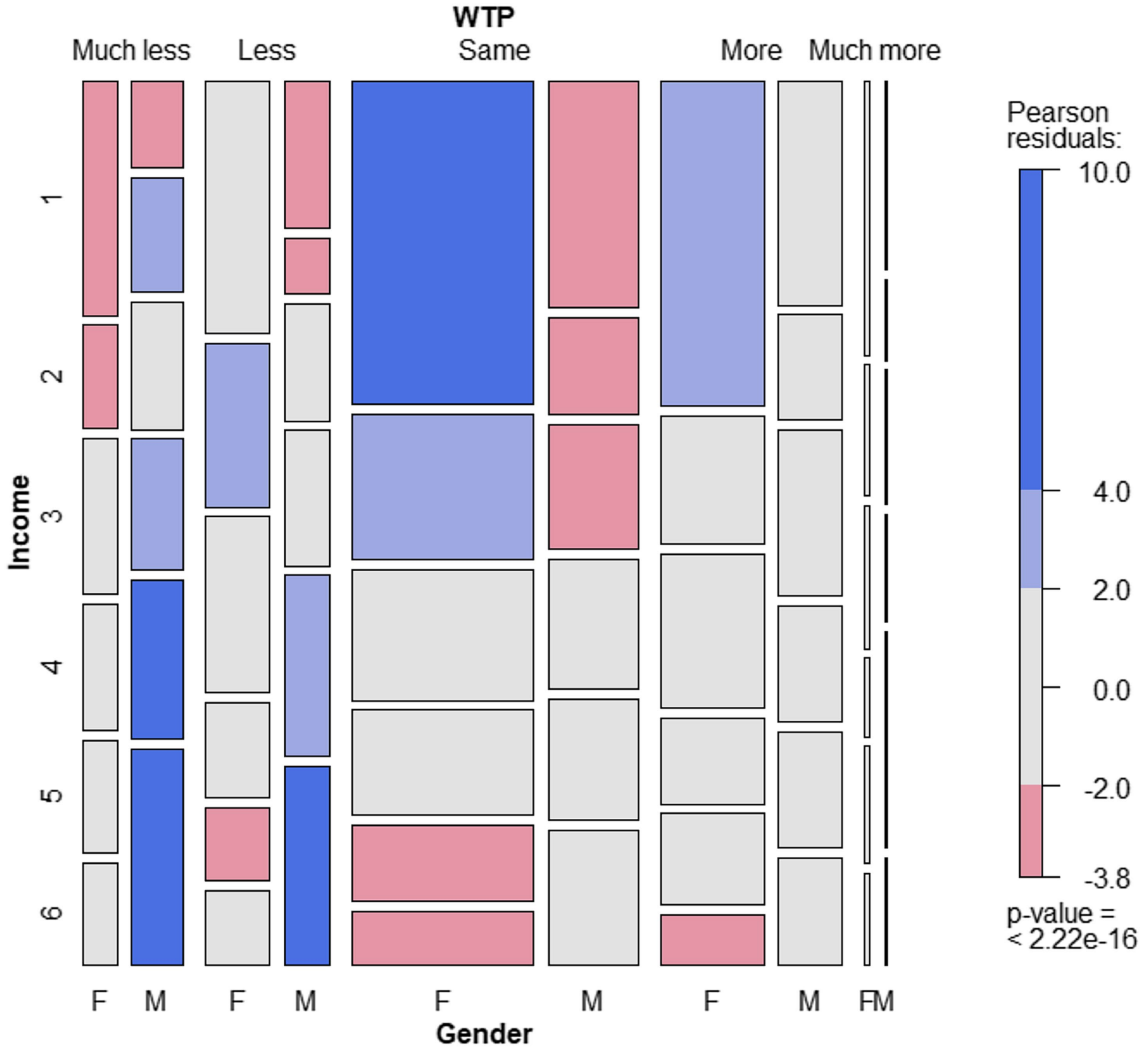
Mosaic plot of the distribution of responses about willingness to pay (WTP) by gender and income (*n* = 3,095). WTP, willingness to pay; Much less = Much less than conventional meat, even nothing at all; Much more = Much more than conventional meat; Less = Less than conventional meat; Same = Same price as conventional meat; More = More than conventional meat. Income: 1 = < €1,500; 2 = €1,500 – €2000; 3 = €2,000 – €2,500; 4 = €2,500 – €3,000; 5 = €3,000 – €4,000; 6 = > €4,000.

Figure 2 provides a detailed overview of the variable WTP in relation to the most significant pair of variables (Gender x Income, *p* = 0.000061). Obviously, in majority, women with a low income (< €1,500) would like to pay the “same” for AM as for conventional meat (or more to a smaller extent). On the other hand, males were willing to pay “much less” or “less” if they belonged to the highest income classes over €3,000.

### 3.5 Driving factors of willingness to engage with AM

**Figure 3:**
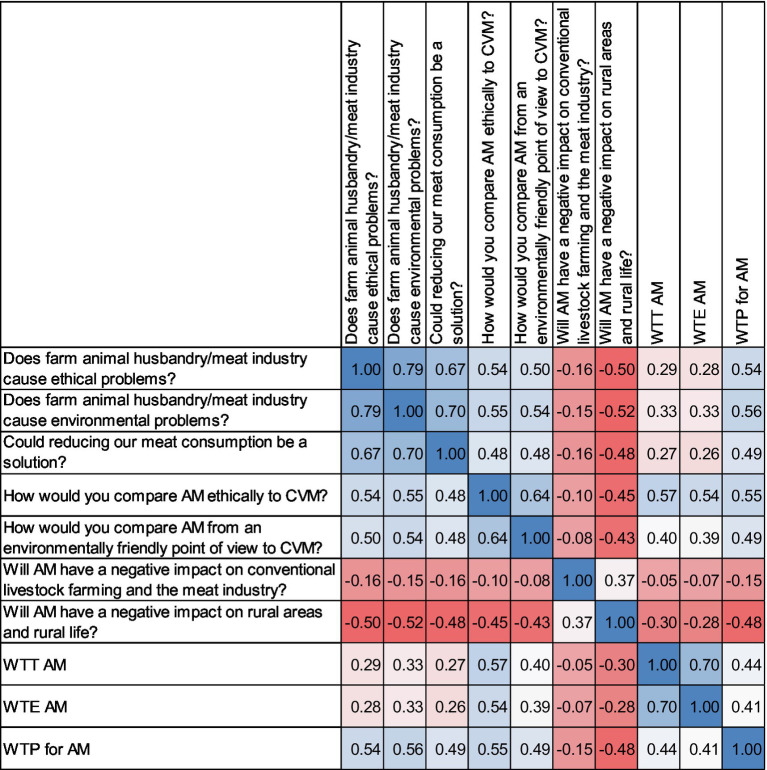
Correlation matrix plot showing the most important correlations between societal challenges and WTT, WTE as well as WTP. Spearman-Rho: All correlations are significant at the 0.01 level (two-sided). Positive correlation = blue; negative correlation = red. The more intense the color, the stronger the correlation. WTE, willingness to eat regularly; WTT, willingness to try; WTP, willingness to pay; AM, artificial meat; CVM, conventional meat.

The results show the most important correlations between societal challenges and WTT, WTE as well as WTP in different color shades (Figure 3). The highest positive correlation was between claims that conventional livestock farming and the meat industry caused environmental problems as well as ethical problems (*r* = 0.79). Reducing meat consumption was seen as a solution to the environmental (*r* = 0.70) and ethical problems (*r* = 0.67) in livestock farming and in the meat industry. Aspects of environmental friendliness and the ethical acceptability of AM compared to conventional meat also had a high positive correlation (*r* = 0.64). In addition, WTE and WTT were positively correlated (*r* = 0.70). How ethical AM would be compared to conventional meat was also positively correlated with WTT (*r* = 0.57).

**Figure 4:**
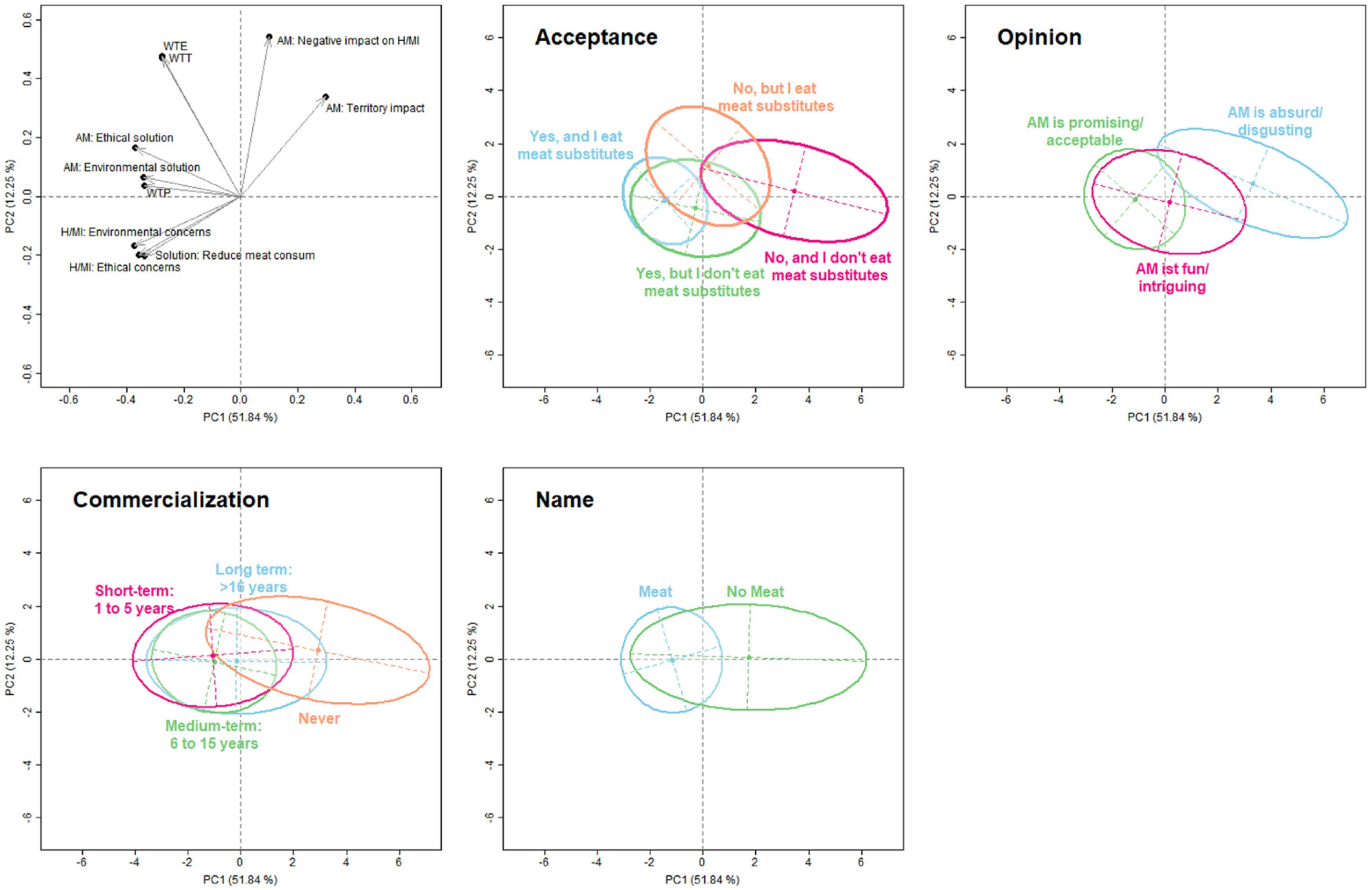
Relationships between variables of positive drivers, motives, barriers and consumer willingness regarding artificial meat. Variables used for the PCA: H/MI: Ethical concerns = “Does farm animal husbandry/meat industry cause ethical problems?”; H/MI: Environmental concerns = “Does farm animal husbandry/meat industry cause environmental problems?”; Solution: Reduce meat consum. = “Could reducing our meat consumption be a solution?”; AM: Ethical solution = “How would you compare AM ethically to CVM?”; AM: Environmental solution = “How would you compare AM from an environmentally friendly point of view to CVM?”; AM: Negative impact on H/MI = “Will AM have a negative impact on conventional livestock farming and the meat industry?”; AM: Territory impact = “Will AM have a negative impact on rural areas and rural life?”; WTT = WTT AM; WTE = WTE AM; WTP = WTP for AM; H, farm animal husbandry; MI, meat industry; AM, artificial meat; WTE, willingness to eat regularly; WTT, willingness to try; WTP, willingness to pay; CVM, conventional meat.

The future of AM in terms of its commercialization, acceptance, naming and general opinion toward it were analyzed (Figure 4). First, the variables related to “social challenges” as well as WTT, WTE and WTP were summarized into principle components (PCs). Figure 4 contains the biplot of this analysis with the projection of the variables. The arrows indicate which variables are highly correlated with PC1 or PC2.

The group of “H/MI: Ethical concerns,” “H/MI: Environmental concerns” and “Solution: Reduce meat consum.” negatively correlated with PC2. In particular, “AM: Environmental solution” and “WTP” had a high positive correlation with PC2. It can also be seen that “AM: Negative impact on H/MI” and “AM: Territory impact” are positively correlated with PC1, with the variable “AM: Negative impact on H/MI” being more closely correlated.

Additionally, Figure 4 shows that optimists ate meat alternatives, accepted AM as such and considered AM to be “acceptable and/or promising,” realistic in 6–15 years and would describe it as meat. Hesitant respondents accepted AM but did not eat meat substitutes, tended to find AM “fun and/or intriguing” but thought it would take a long time before it was released to the market. Skeptics or opponents did not eat meat substitutes, would certainly not accept AM and considered it “absurd and/or disgusting.” The idea that AM could take over the food market and be labeled as meat was a major obstacle for them.

### 3.6 Relationships among respondents’ attitudes, perspective, acceptance, and willingness to try (WTT) and to eat (WTE) AM regularly

**Figure 5:**
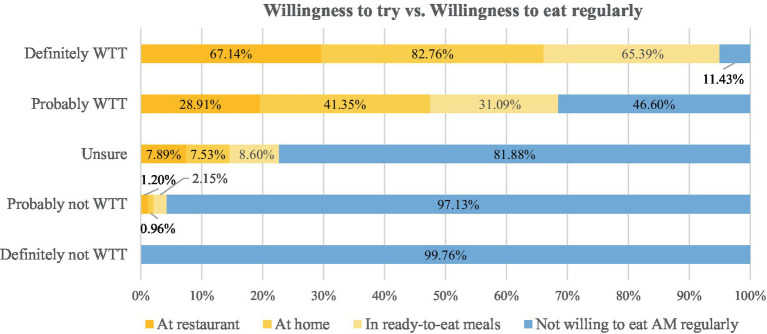
Cross analysis between willingness to try (WTT) and willingness to eat (WTE) artificial meat regularly at restaurant, at home or in ready-to-eat meals (in %; multiple choice). WTT, willingness to try; WTE, willingness to eat regularly; AM, artificial meat.

The results in Figure 5 initially show the cross analysis between WTT and WTE in different settings. It is particularly noteworthy that of the respondents who had “probably” or “definitely” no WTT, also 99.8 and 97.1%, respectively, would not eat it regularly. However, of those who were “unsure” about WTT, the vast majority (81.9%) were certain that they would not like to eat it regularly. Among those who were “definitely” willing to try, 82.8% wanted to eat it regularly at home, 67.1% at a restaurant and 65.4% in ready-to-eat meals. Only 11.4% of them did not have WTE. This percentage was much higher among those who only “probably” (46.6%) wanted to try AM.

**Figure 6:**
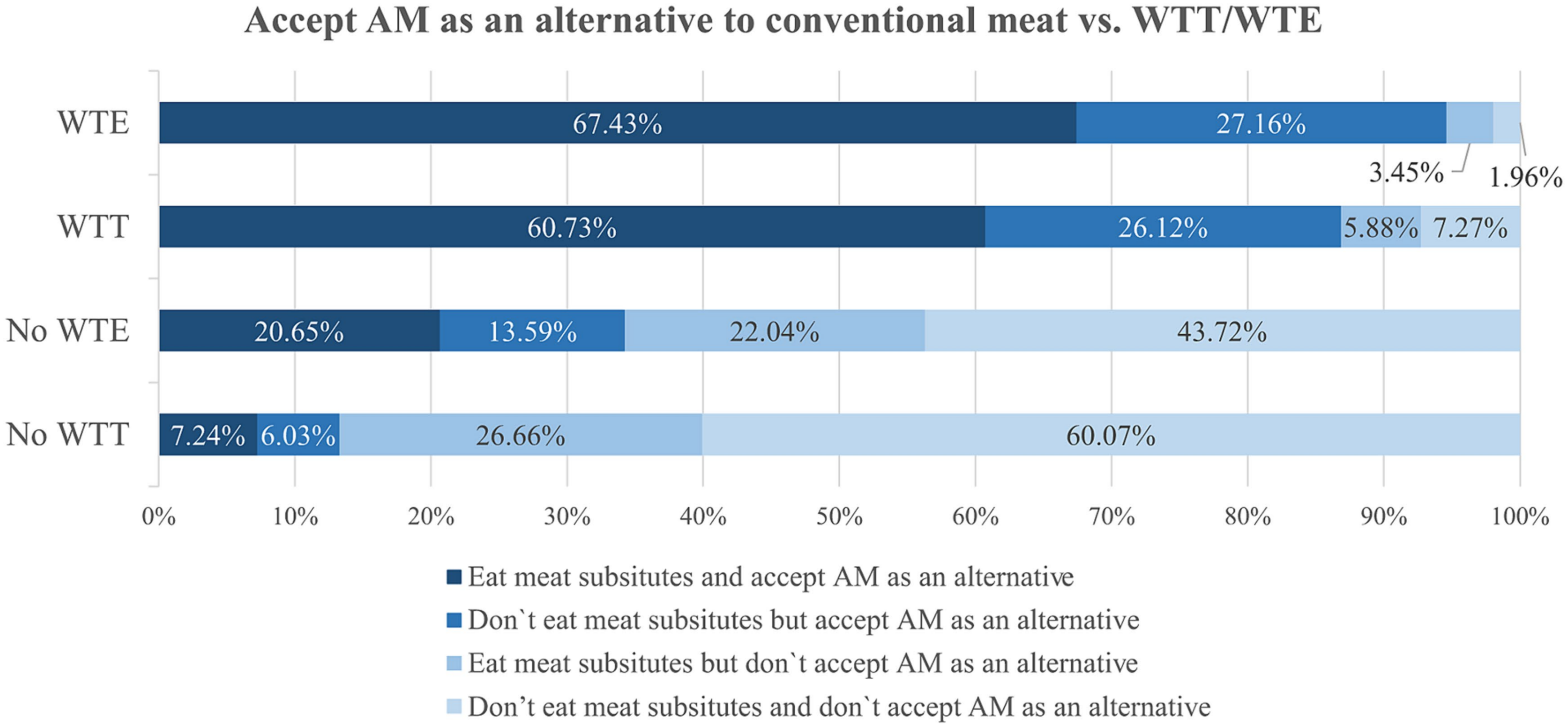
Cross analysis between willingness to try artificial meat (WTT), willingness to eat artificial meat (WTE) regularly and accepting artificial meat as an alternative (in %). WTE, willingness to eat regularly; WTT, willingness to try; WTP, willingness to pay; AM, artificial meat.

A large majority of respondents already ate meat substitutes and would also accept AM as an alternative to conventional meat (67.4 and 60.7% for WTE and WTT, respectively) (Figure 6). On the contrary, regarding respondents with no WTT and WTE, this percentage (21 and 7%, respectively) was much lower.

Most respondents without WTT (60.07%) as well as 43.7% without WTE did not eat meat alternatives and would not accept AM as such (60.07%). Nearly 7.3% of those with WTT and 2% of those with WTE did not eat meat substitutes and would not accept this new product as an alternative to conventional meat.

**Figure 7:**
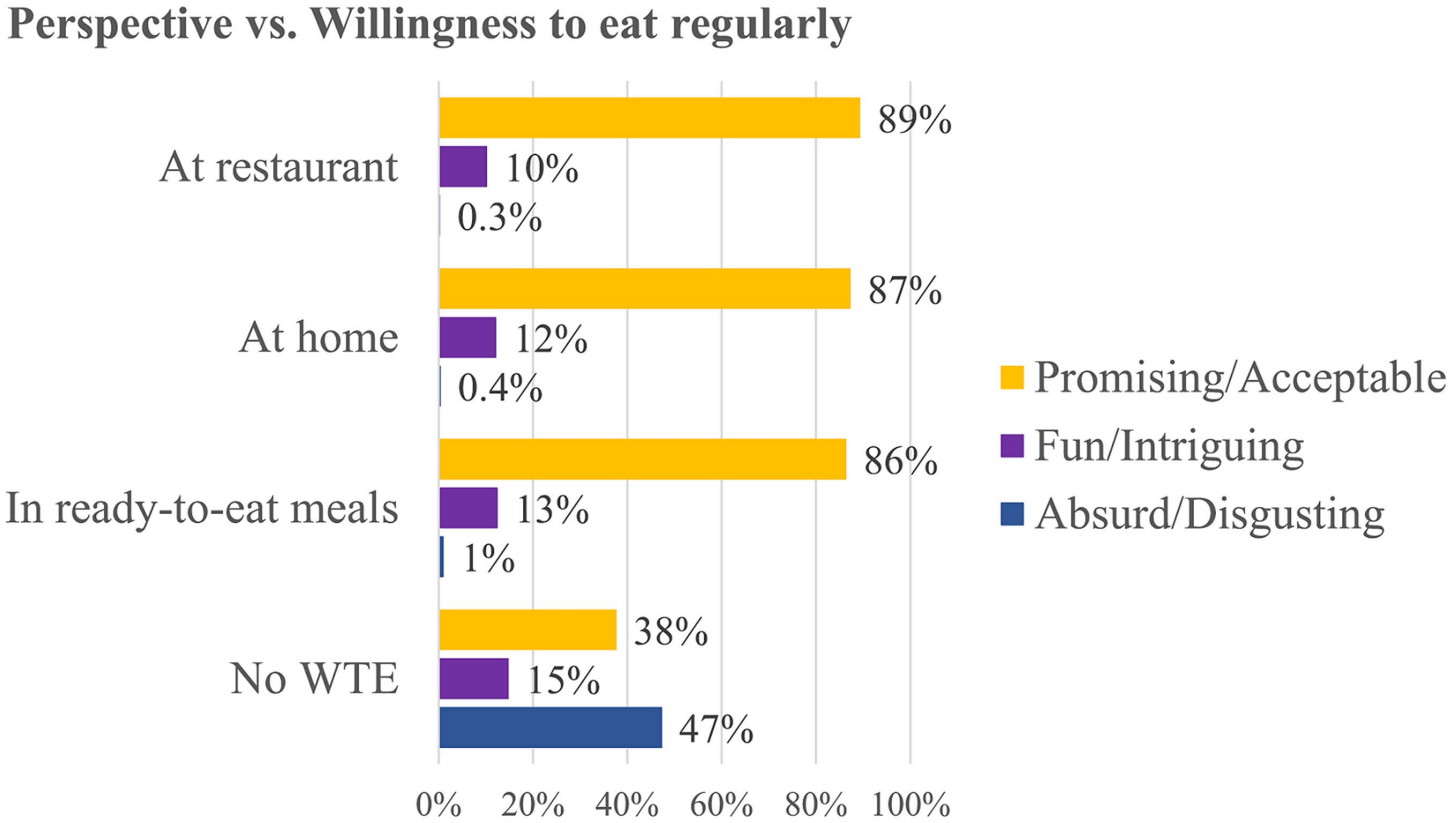
Cross analysis between respondents’ perspective of artificial meat and willingness to eat artificial meat regularly (WTE) in different contexts (in %; multiple choice). AM, artificial meat; No WTE, no willingness to eat AM regularly.

The majority of respondents with WTE and who felt AM was “promising/acceptable” had high percentages for the options of eating it at a restaurant, at home or in ready-to-eat meals (multiple choice; 89, 87, and 86%, respectively) (Figure 7). Participants who found this new product “fun/intriguing” were willing to consume it primarily in ready-to-eat meals (13%), followed by consumption at home (12%) and at a restaurant (10%). Among those who characterized AM as “absurd/disgusting,” WTE scored the lowest (0.3% at restaurant, 0.4% at home and 1% in ready-to-eat meals).

Among those without WTE, a total of 47% think that AM would be “absurd/disgusting,” 15% chose the “fun/intriguing” combination, and a high percentage of 38% perceived it as “promising/acceptable.”

### 3.7 Designation of the new product

**Figure 8:**
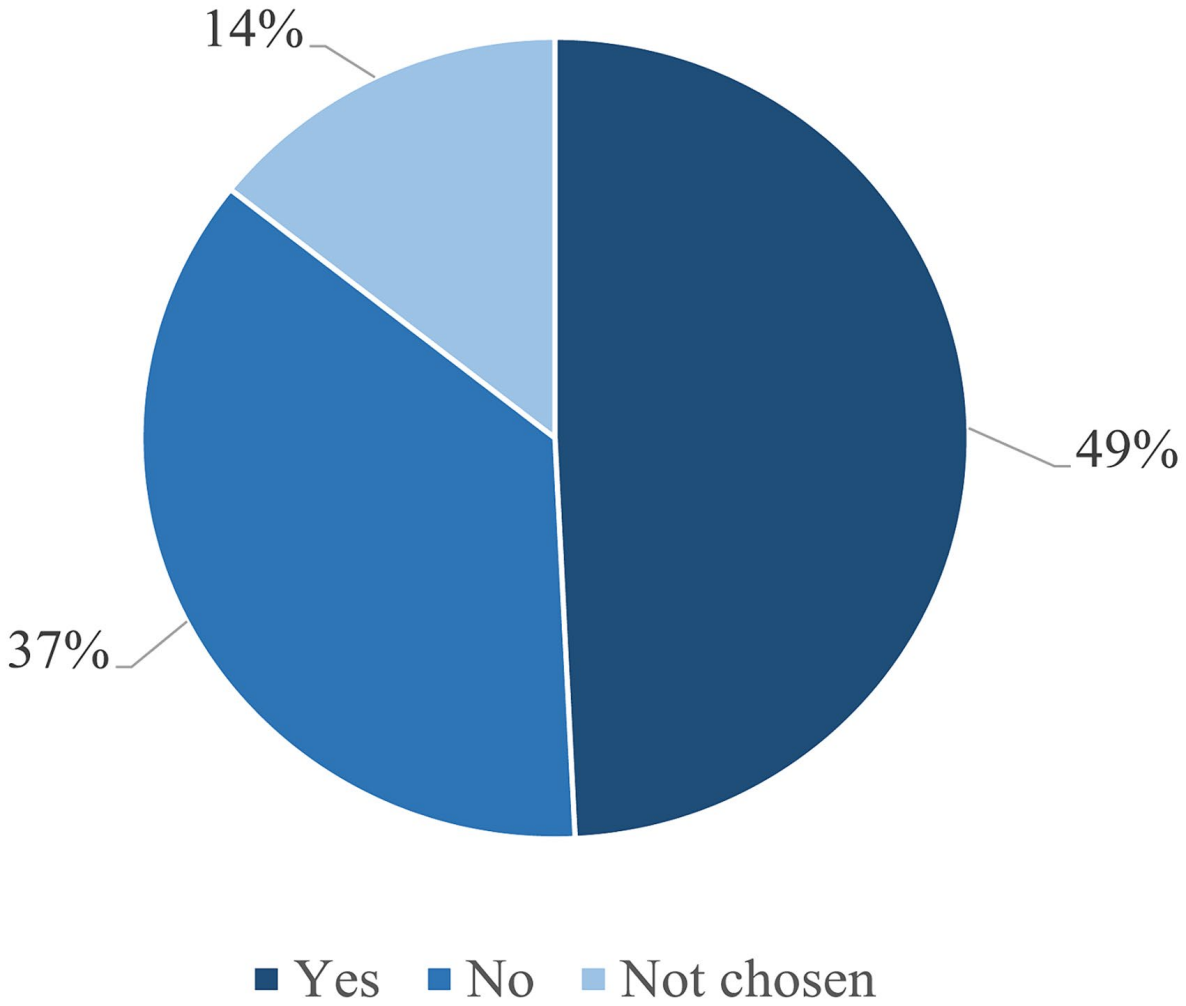
Should this new product be called “meat”? (*n* = 4,713).

The most frequently chosen designations by respondents were “artificial meat” (30%) and “*in vitro* meat” (28%) (Supplementary Table A5). Almost half of the respondents (49.2%) thought that the new product should be called “meat,” 36.4% were against this term and 14.3% did not select any answer at all (Figure 8).

## 4 Discussion

### 4.1 German respondents had a high willingness to engage with AM

Through the evaluation of the 3,558 responses, we confirmed H1a that the participants in this study were predominantly forward-looking and were highly committed to try and eat this new product.

We initially inferred the positive attitude of respondents mainly from the high figures for WTT (70%) and WTE (57%). Additionally, 63% of the respondents considered AM to be a promising/acceptable product. Although the structure of socio-demographic factors varied, high WTT was observed worldwide in analyses from the USA (65 and 66%), Brazil (66%) and France (80%) (17, 27–29).

Compared to our study, Weinrich et al. (30) observed a lower willingness for Germans (57%) and described them as being “unenthusiastic” toward this new product (*n* = 713, representative). The authors also reported a lower level of pre-knowledge among their respondents (38.1%).

The high engagement in this study on AM resulted from the fact that our participants were very familiar with the topic and better informed (95%) than in previous studies. This may be explained by the timeliness of our more recent study and an increasing demand for meat alternatives in Germany, which also results in a wide range of information on future protein supply, especially through the public media. Additionally, the vast majority of our respondents were well educated consumers. Sajdakowska et al. (31) explained that education level may be associated with different levels of knowledge about nutrition. They further summarized that this is one of the most important prerequisites (31), because according to Ronteltap et al. (32) initial knowledge of an innovation constitutes the first step in the innovation decision-making process.

The results were interpreted accordingly considering all our respondents were volunteers to answer to our survey. The fact that our respondents had a previous knowledge of AM suggest that their opinion has been made before the survey and not at the time of the survey.

The most important positive drivers that motivate German respondents to be interested in AM were ethics, curiosity and respect for the environment. It is evident that only one decisive factor for the absence of WTT did not exist among the German participants, with almost similar percentages for: unnaturalness, feeling of dislike, less trust in companies and less tastiness. The fact that the German respondents were well-informed in advance could also mean that they were more aware of the potential multifactorial challenges of AM.

Further results in our study underline the great importance of a clear and positive attitude toward WTT and WTE of this new product, which confirms H1b (positive opinions about AM will have a very positive impact on WTT and WTE). Once German study participants answered “definitely” for WTT, their dislike for WTE was almost four times lower than those respondents who answered “probably” for WTT. In this respect, the Chinese were somewhat inconsistent, as more consumers could imagine eating the product regularly even if they had no WTT (18).

Although sales of meat alternatives are increasing in Germany, our study does not reveal a clear acceptance of AM as such a product. Among the respondents who had previously eaten meat alternatives, not even half of them (46%) accepted AM as such a product, which seems somewhat surprising at first. However, this acceptance increased when WTT and WTE correlated with respondent’s willingness to eat meat alternatives (+15% and + 21%, respectively). From this, one can cautiously conclude that AM was seen as a meat product rather than an alternative like plant-based substitutes. We can confirm this hypothesis by the fact that if AM could be purchased in future, almost half of the participants (49.2%) said it should be called “meat.”

### 4.2 AM was mostly attractive to younger German consumers, especially young men

Regarding H2, the findings of this study verified important associations between significant demographic factors and WTT or WTE.

With increasing age, WTT and WTE of respondents decreases, regardless of indicated gender. Young men as well as men with low income or rare meat consumption had the highest WTT and WTE. According to a study by Wilks, and Phillip (29) as well as that by Liu et al. (18), American and Chinese men, respectively, were also more willing to eat AM than female respondents. However, in the Chinese analysis, older males (≥ 31 years of age) had high WTT, which contrasted with the German study on this point. This is also true for Brazilian results in which young females had the highest WTT (17).

One reason for men’s higher interest in AM in this study may be that meat or sausage were much more likely to be on a man’s menu at least once a day (33%) than on a woman’s menu (18%) (33). German men also believed more than women that food in Germany is safe (33). This may lead to some openness and trust in new products like AM among men.

Furthermore, based on age, young consumers with a high level of education (PhD degree) had the highest WTT, while those with incomes up to €3,000 had the highest WTE. This could be related to the fact that, in Germany, for the under 30 age group, those with the highest salaries do not earn as much as other age groups (34).

Meat consumption was one of the deciding factors for engaging with AM in this study. The highest willingness for WTT and WTE was found among respondents with a rarely (weekly or less) meat consumption. In particular, respondents aged 18–30 with a rarely meat consumption had the highest WTE.

It can be assumed that people who only eat meat once a week or even less often eat a so-called “flexitarian” diet. Their diet focuses on plant-based products, but they would like to consume meat on special occasions in particular and not avoid it completely (35). This is a growing trend in Germany and both the demand and the supply of products for this kind of diet are increasing (35). This may explains why the respondents with a rarely meat consumption in our study were particularly interested in AM.

Therefore, it was found that high daily/regular meat consumption was not associated with greater WTT and WTE, particularly among older males (≥ 51 years of age) and young consumers. This trend is almost similar to that of French respondents (36). However, these results do not match other studies by Liu et al. (18), Gousset et al. (28), and Bryant et al. (37) which analyzed a higher level of skepticism among vegetarians and vegans.

Considering that ethical issues were the most important aspects for WTT among all German respondents, it can be also cautiously concluded that AM was a more ethical solution than traditional meat dishes in the eyes of those German participants who rarely or never eat meat. Gousset et al. (28) indicated that people who do not consume animal products tend to view AM as a throwback to the past. In any case, our results reinforce the perception that especially younger consumers are particularly interested in how protein, meat and its alternatives will be produced in the future.

Although the respondents did not reflect the German population in all respects, their demographic structure can be counted among the so-called earlier adopters (women / high income respondents / young people / well-educated consumers). Thus, a comparison with those who adopt innovations or so-called trendsetters is given. This is an important aspect as it can play a major role in the course of diffusion and commercialization of this new product and the development of conventional meat production.

### 4.3 German consumers were sensitive to the price of this new product

For almost half of the German respondents, price played a major role when purchasing food (Supplementary Table A6), which was consistent with for just under half of Germans in 2021 (33).

The well-known price sensitivity in Germany could be observed, as the vast majority of the participants were not in favor of a higher price than for conventional meat (lower price: 33%; same price: 40%) that confirmed H3.

French consumers were also more reluctant to pay a higher price (68.5%) (36) as were African consumers (nearly 61%) (16). It can reasonably be assumed that AM will not be in the higher price segment for the broad customer based on the German food market; especially if it was presented as an equivalent to conventional meat. Here, there must at least be a positive benefit of AM, such as ethics or environmental friendliness.

However, WTP strongly depends on sociodemographic factors. Young German consumers with a high level of education or income up to €3,000 as well as consumers who did not eat meat had a high WTP. Young female respondents in particular had a slightly higher WTP. However, it should be noted that the mean values for a high WTP were close to score 3, meaning that respondents were only willing to pay almost the “same” for AM as for traditional meat.

In general, German men and women paid almost equal attention to price when choosing food (13); in this respect, our study is different. The analysis showed that particularly wealthy males were willing to pay “much less/less” for AM while women with low incomes would like to pay the “same,” but also not more. Our results are consistent with the findings from Brazil (17). These results seem to be counter-intuitive, as one would expect that wealthy people would initially be comfortable paying for an innovation or a new product. Nevertheless, in Germany, women were significantly more likely than men to say that they bought alternative products to meat for animal welfare, environmental and climate protection reasons, and they paid more attention on local sourcing of their food (33). This also applied to those under the age of 30 (33).

The results of Chriki et al. (17) for Brazilian consumers also confirmed higher WTP among younger people, especially young women.

Hocquette et al. (36) found that young French people had the highest WTP when they did not eat meat, unlike older people with daily meat consumption. Similarly, our vegetarian and vegan participants, regardless of gender, age and income, had the highest WTP compared to meat eaters. Generally speaking, the characteristics of a vegetarian/vegan diet include above all ethical aspects and therefore vegans reject any food of animal origin including AM which does indeed come from animal cells (38). In this respect, the higher WTP among vegetarians and vegans in our study could be explained by the fact that they perceived this novel product as an innovation but did not expect to buy it because it would still be meat. In addition, they were accustomed to high product prices for alternative meat products such as plant-based ones in Germany (39). In Germany, typical vegetarians and vegans were predominantly female, young and well-educated (33, 38, 40). However, the higher education level of vegans compared to the general population is not necessarily accompanied by a higher household income (38). This to some extent explains the above results of women with low income but a high WTP. In general, respondents´ expectations regarding sanitary, nutritional and sensory quality of AM were not excessively high.

### 4.4 AM was perceived as a solution to ethical and environmental problems

Regarding H4, we found that AM was perceived as a solution to ethical and ecological problems. In particular, our study participants expected a AM production without animal suffering (68%) and with a smaller ecological footprint (61%) (multiple choice) which was consistent with the summarized results of Bryant and Barnett (41).

Although claims that conventional livestock farming and the meat industry caused “environmental problems” as well as “ethical problems” are closely related, the German respondents believed in the future of the current agricultural system. Only 6% of them assumed a complete future abandonment of conventional agriculture and 20% of them suspected a decline thanks to AM. These statements showed the interviewees’ awareness of possible transformative aspects of German agriculture and food processing.

The main so-called positive drivers and motives for German consumers’ engagement with AM underlined this. These included the problems of traditional farming, reducing meat consumption as a solution to this problem and the higher ecological/ethical acceptance of AM compared to conventional meat. The assessment by the German respondents was consistent with analyses and opinions that AM production is a growing industry that promises to use less water and land or to reduce air pollution and emission of greenhouse gases [e.g. (42–44)]. However, AM is not necessarily a more sustainable alternative compared to beef or poultry meat production (44, 45). It often depends on many different aspects of the inputs and this new product only has a lower environmental impact in the best-case scenarios (44). In any case, according to SINUS Markt- und Sozialforschung (46), sustainability has become a guiding principle in Germany; we can also speak of a social norm. Sustainability is now only a question of “how” and not “if” (46).

### 4.5 AM in Germany was considered realistic, but not in the very near future

Almost 75% of the respondents envisaged AM production and consumption in Germany in more than five years and only 6% in the very near future (< 5 years) despite huge investments and advertising in public media. With these results, we could confirm H5. In addition, 20% did not expect this new product to come to fruition. The results of the Brazilian respondents are almost consistent with our data (17). As our analysis showed, different factors influenced the marketing conditions, acceptance, and naming of AM and the respondents evaluated the future of AM differently. These finding focused on three main personality types for German consumers: optimists, hesitant ones/expectants, and skeptics.

For future research in the field of AM, respondents considered both a private research model and responsibility of public research to be relevant. According to a report commissioned by the German federal government, targeted funding of research in the field of AM and, in particular, support for the transfer of science to practice will strengthen Germany’s position in the market (47).

### 4.6 Limits and strengths of the survey

Neutrality in this survey was ensured by the University of Veterinary Medicine Hannover, the wording of the questions and the structure of the questionnaire (17).

The results of our web survey are “in principle not generalizable,” as they were based on the random principle of who would answer, and not on a predetermined population (48). However, we collected important demographic factors of the respondents which could thus be evaluated and compared to the demographic characteristics of the German population. This was also the approach taken in previous studies by Chriki et al. (17), Hocquette et al. (19), and Liu et al. (18).

Compared to the social-demographic structure of the adult German population (49–52), our sample was not representative in all points. The proportion of female respondents in this study was slightly higher than in the German population, where the proportion of men and women is almost identical. The high proportion of younger participants can be explained in particular by their interest in healthy and animal-free nutrition in Germany or because it was an online survey. One reason for the higher educational qualification of our respondents can be seen in the distribution of the survey as it was published on the university’s Facebook page, which has a large audience in the highly qualified sector. It can be cautiously concluded that the higher level of education was associated with a higher income among the respondents.

The higher level of education and the slightly higher proportion of women in our study compared to the German population could be an explanation for the higher proportion of vegetarians and vegans among our respondents (30%), as these characteristics are often associated with choosing this diet in Germany (33, 38, 40).

As often acknowledged, the results of surveys on AM may be biased because the product is not currently available on the market (53). To the best of our knowledge, however, this study is one of the quantitatively largest surveys performed in Germany about consumers’ potential attitudes toward AM.

### 4.7 Term “artificial meat”

It must be pointed out that we used the term “Künstliches Fleisch” (“artificial meat”) in our German questionnaire. In this context, the choice of the term for the new product can provoke negative as well as positive emotions (30). Analysis of the FAO & WHO (54) found out that especially in recent years, the term “cultured meat” has been used most frequently in scientific research as well as in the media and other sectors. However, it seems that the participants did not perceive the term selected in this study very negatively, as they had a high willingness to engage with the new product. In addition, the most frequently chosen designations by respondents for the new product were “artificial meat” and “*in vitro* meat,” although the term “artificial meat” was used in the questionnaire and may therefore have caused a habituation effect.

## 5 Conclusion

Due to the high number of German adults participating in the survey (*n* = 3,558), important information regarding the acceptance and future development of AM in Germany could be obtained. Age, income, meat consumption and education level were drivers of interest in AM. From these criteria, specific characteristics of the type of consumers in Germany were drawn. It can be assumed that these consumers could be identified as early adopters of this potentially new product.

Furthermore, the future willingness to try, eat and recognize AM as an alternative product to conventional meat will be a very important prerequisite for regular consumption and acceptance if it is launched on the market. Overall, AM is a complex issue, as this product is not yet available on the German food market. In general, the success of innovations depends on many factors. Some of these innovations cannot stay on the market for very long because the dynamics and the new products are too innovative to be accepted by the general public. However, as the results of this study show, AM could be more of a disruptive innovation, as acceptance of trying and eating it were very high. However, in this context, is the fact that only a few participants envisaged a complete future abandonment of traditional agriculture because of AM. WTP for AM in Germany can also be described as rather price-sensitive. Key factors for the diffusion and commercialization of this new product in Germany will certainly be the aspects related to climate friendliness, price and the acceptance of the product as an alternative to traditional meat. In conclusion, the results of this study are important to discuss a paradigm shift in global meat production with respect to sustainability, food safety, demand for meat and the potential adoption of new food products.

## Data availability statement

The original contributions presented in the study are included in the article/Supplementary material, further inquiries can be directed to the corresponding author.

## Ethics statement

This study is part of an international survey (available in different languages including Arabic, Chinese, English, French, Italian, Portuguese and Spanish) which was conducted following local guidelines based on the laws and regulations of the countries in which the research was performed (in this case, based on practices of Germany, University of Veterinary Medicine Hannover (TiHo), Foundation) and including ethical approval by ethics committees if required (such as in Brazil with number CAAE: 37924620.5.0000.5404).

## Author contributions

A-KJ: Data curation, Formal analysis, Investigation, Methodology, Project administration, Resources, Supervision, Validation, Visualization, Writing – original draft, Writing – review & editing. H-WW: Project administration, Resources, Supervision, Writing – original draft, Writing – review & editing. JG: Formal analysis, Methodology, Resources, Visualization, Writing – review & editing. SC: Conceptualization, Methodology, Project administration, Resources, Writing – review & editing. J-FH: Conceptualization, Methodology, Project administration, Resources, Writing – original draft, Writing – review & editing. M-PE-O: Conceptualization, Methodology, Project administration, Resources, Supervision, Validation, Visualization, Writing – original draft, Writing – review & editing.
