# Supplementary material for: German consumers’ attitudes toward artificial meat
Source: Front Nutr. 2024 Jun 12;11:1401715. doi: 10.3389/fnut.2024.1401715 (PMC11199728; doi:10.3389/fnut.2024.1401715)
Supplement: Supplementary file 1 [file Data_Sheet_1.pdf]

Supplementary material

German consumers' attitudes towards artificial meat

Anne-Katrin Jacobs[1*], Hans-Wilhelm Windhorst[1,2], Julia Gickel[1], Sghaier Chriki[3,4], Jean-François Hocquette[3], Marie-Pierre Ellies-Oury[3,5]

[1]Science and Innovation for Sustainable Poultry Production (WING), University of Veterinary Medicine Hannover (TiHo), Foundation, Field Station for Epidemiology (Bakum), Büscheler Straße 9, 49456 Bakum, Germany
[2]University of Vechta, Department II - Natural and Social Sciences, Driverstraße 22, 49377 Vechta, Germany
[3]Université Clermont Auvergne, Institut National de Recherche pour l'Agriculture, l'Alimentation et l'Environnement (INRAE), VetAgroSup, UMR1213, Recherches sur les Herbivores, Theix, 63122 Saint-Genès Champanelle, France
[4]Bordeaux Sciences Agro, Département Feed and Food, 33175 Gradignan Cedex, France
[5]Isara, 23 rue Jean Baldassini, 69364 Lyon, France

*Correspondence:
Anne-Katrin Jacobs
anne-katrin.jacobs@tiho-hannover.de

Supplementary Figure A1. Copy of the questionnaire “Umfrage zu künstlichem Fleisch“ (“Survey on artificial meat”).

Umfrage zu künstlichem Fleisch

Einleitung und Kontext der Umfrage

Künstliches Fleisch, auch bekannt als In-vitro-Fleisch, kultiviertes Fleisch, Laborfleisch, Clean Meat oder auch synthetisches Fleisch, ist ein sogenanntes Novel-Food (Neuartiges Lebensmittel), welches mithilfe von tierischen Muskelstammzellen in einer Nährlösung vermehrt wird, wobei die Zellen nicht direkt vom Tier stammen.

Die Produktion von künstlichem Fleisch ist Gegenstand eines medialen Enthusiasmus, der sich vor allem auf die wachsende Bevölkerung und die dadurch steigende Nachfrage nach Lebensmitteln bezieht. Um den zunehmenden Problemen hinsichtlich der Umwelt (globale Erwärmung) und ethischen Belangen (Tierwohl, Tierleid und Schlachtung), aber auch den Herausforderungen der konventionellen Fleischerzeugung (begrenzte landwirtschaftliche Ressourcen und eine stetig wachsende Bevölkerung) zu begegnen, muss sich auch die Wissenschaft in großem Maße der Einführung und Entwicklung von künstlichem Fleisch als zukünftiges neues Fleischprodukt widmen.

Diese Studie wird von den französischen Wissenschaftlern der INRAE (Theix, 63), ISARA (Lyon, 69) und Bordeaux Sciences-Agro (Bordeaux, 33) durchgeführt. Die Forschungseinrichtung WING (Wissenschaft und Innovation für Nachhaltige Geflügelwirtschaft) der Stiftung Tierärztliche Hochschule Hannover unterstützt die Studie hinsichtlich der Ansprache deutscher Verbraucherinnen und Verbraucher.

Das Ziel der Studie ist es, Meinungen von Konsumenten zu dieser neuen Biotechnologie abzufragen und ihre Vorlieben und/oder Abneigungen zu erheben. Sie haben die Möglichkeit, an diesem Forschungsprojekt mitzuwirken, indem Sie an einer Online-Befragung teilnehmen. Diese sollte nicht mehr als 15 Minuten Ihrer Zeit in Anspruch nehmen. Gemäß dem Ethikkodex der Umfrageinstitutionen und den rechtlichen Anforderungen an den Datenschutz stellen wir sicher, dass Ihre persönlichen Daten und die von Ihnen bereitgestellten Angaben vertraulich behandelt, datenschutzkonform verarbeitet und nur im Rahmen der statistischen Auswertungen der Studie verwendet werden.

Um an dieser Umfrage teilnehmen zu können, sollten Sie das 18. Lebensjahr vollendet haben, darüber hinaus benötigen Sie keine speziellen Kenntnisse oder Fachwissen. Bitte lesen und akzeptieren Sie die Datenschutzerklärung und klicken Sie auf die Schaltfläche "Weiter", um Zugang zur Umfrage zu erhalten und automatisch der Teilnahme zuzustimmen. Es ist zu jedem Zeitpunkt möglich, die Befragung abzubrechen. In diesem Fall werden bereits angegebene Informationen NICHT gespeichert. Dies wird alleinig durch die Zustimmung am Ende der Befragung gewährleistet.

Hinweis: Am besten führen Sie die Umfrage am Computer oder Laptop durch, da die Darstellung hierfür optimiert wurde.

Bei Anliegen und Fragen melden Sie sich bitte per E-Mail unter: futurecellmeat@gmail.com oder für Deutschland bei anne-katrin.jacobs@tiho-hannover.de

Prinzipien der Herstellung von künstlichem Fleisch

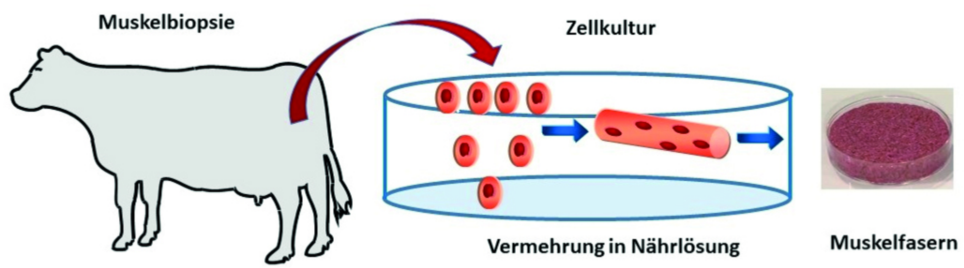

Bei allen mit * gekennzeichneten Fragen sind Angaben erforderlich

In dieser Umfrage sind 33 Fragen enthalten.

Figure A1. Cont.

Bei allen mit * gekennzeichneten Fragen sind Angaben erforderlich

In dieser Umfrage sind 33 Fragen enthalten.

Demographische Daten

1. Geschlecht *

Bitte wählen Sie eine der folgenden Antworten:
Bitte wählen Sie nur eine der folgenden Antworten aus:

- ◯ Weiblich
- ◯ Männlich
- ◯ Möchte darauf nicht antworten

2. Land oder Kontinent der Herkunft *

Bitte wählen Sie eine der folgenden Antworten:
Bitte wählen Sie nur eine der folgenden Antworten aus:

- ◯ Deutschland
- ◯ Andere Länder innerhalb der Europäischen Union
- ◯ Europäische Länder außerhalb der Europäischen Union
- ◯ China
- ◯ Russland
- ◯ Mittlerer Osten und Asien (ausgenommen China und der asiatische Teil Russlands)
- ◯ Nordamerika
- ◯ Südamerika
- ◯ Afrika
- ◯ Ozeanien

3. Alter *

Bitte wählen Sie eine der folgenden Antworten:
Bitte wählen Sie nur eine der folgenden Antworten aus:

- ◯ 18-30 Jahre alt
- ◯ 31-50 Jahre alt
- ◯ 51 Jahre alt und älter

4. Bildungsabschluss *

Bitte wählen Sie eine der folgenden Antworten:
Bitte wählen Sie nur eine der folgenden Antworten aus:

- ◯ Hauptschulabschluss oder Realschulabschluss
- ◯ (Fach-)Abitur
- ◯ Hochschulabschluss
- ◯ Doktortitel
- ◯ Ich möchte nicht antworten
- ◯ Sonstiges

5. Tätigkeitsgebiet *

Bitte wählen Sie eine der folgenden Antworten:
Bitte wählen Sie nur eine der folgenden Antworten aus:

- ◯ Wissenschaftler innerhalb des Fleischsektors
- ◯ Wissenschaftler außerhalb des Fleischsektors
- ◯ kein Wissenschaftler aber Tätigkeit innerhalb des Fleischsektors
- ◯ kein Wissenschaftler und Tätigkeit außerhalb des Fleischsektors

6. Monatliches Nettoeinkommen

*

Bitte wählen Sie eine der folgenden Antworten:
Bitte wählen Sie nur eine der folgenden Antworten aus:

- ◯ 1.500 € oder weniger
- ◯ 1.500 – 2.000 €
- ◯ 2.000 – 2.500 €
- ◯ 2.500 – 3.000 €
- ◯ 3.000 – 4.000 €
- ◯ 4.000 € oder mehr
- ◯ Ich möchte nicht antworten

7. Fleischkonsum *

Bitte wählen Sie eine der folgenden Antworten:
Bitte wählen Sie nur eine der folgenden Antworten aus:

- ◯ Nie: Vegetarische oder vegane Ernährung
- ◯ Selten: Wöchentlich oder weniger
- ◯ Regelmäßig: Mehrmals wöchentlich
- ◯ Täglich oder zu jeder Mahlzeit

Einführung

8. Haben Sie schon einmal von künstlichem Fleisch gehört? *

Bitte wählen Sie eine der folgenden Antworten:
Bitte wählen Sie nur eine der folgenden Antworten aus:

- ◯ Ja
- ◯ Nein

9. Was sind die wichtigsten Kriterien während Ihres Lebensmitteleinkaufs? *

Bitte wählen Sie die zutreffenden Antworten aus:
Bitte wählen Sie alle zutreffenden Antworten aus:

- ☐ Ethik (Haltungssysteme, Produktionsmethoden, Tierwohl…)
- ☐ Umweltbelastung
- ☐ Preis
- ☐ Sensorische Qualität (Geschmack, Saftigkeit, Zartheit…)
- ☐ Aussehen
- ☐ Energieaufnahme (Kalorien)
- ☐ Nährwert (Proteine, Vitamine…)
- ☐ Herkunft und Rückverfolgbarkeit
- ☐ Sicherheit
- ☐ Marken und/oder Labels
- ☐ Sonstiges:

Figure A1. Cont.

Gesellschaftliche Herausforderungen

10. Denken Sie, dass die Nutztierhaltung und Fleischindustrie für ethische Probleme sorgen (z. B. Tierleid, Schlachtung...)?

1 = Wesentlich weniger / Stimme gar nicht zu
2 = Weniger / Stimme nicht zu
3 = Neutral / Stimme weder zu noch nicht zu
4 = Mehr / Stimme zu
5 = Wesentlich mehr / Stimme absolut zu

*

Bitte wählen Sie maximal eine Antwort.
Bitte wählen Sie die zutreffende Antwort für jeden Punkt aus:

1	2	3	4	5
○	○	○	○	○

11. Denken Sie, dass die Viehzucht und die Fleischindustrie für Umweltprobleme sorgen (z. B. hoher Wasserverbrauch, Treibhausgasemissionen...)?

1 = Wesentlich weniger / Stimme gar nicht zu
2 = Weniger / Stimme nicht zu
3 = Neutral / Stimme weder zu noch nicht zu
4 = Mehr / Stimme zu
5 = Wesentlich mehr / Stimme absolut zu

*

Bitte wählen Sie maximal eine Antwort.
Bitte wählen Sie die zutreffende Antwort für jeden Punkt aus:

1	2	3	4	5
○	○	○	○	○

12. Um diesen potentiellen Problemen entgegenzuwirken, denken Sie, dass die Reduzierung unseres Fleischkonsums eine Lösung sein könnte?

1 = Wesentlich weniger / Stimme gar nicht zu
2 = Weniger / Stimme nicht zu
3 = Neutral / Stimme weder zu noch nicht zu
4 = Mehr / Stimme zu
5 = Wesentlich mehr / Stimme absolut zu

*

Bitte wählen Sie maximal eine Antwort.
Bitte wählen Sie die zutreffende Antwort für jeden Punkt aus:

1	2	3	4	5
○	○	○	○	○

13. Was denken Sie, wie ethisch vertretbar künstliches Fleisch gegenüber konventionellem Fleisch wäre (z. B. in der Lage, Tierwohl zu verbessern, Tierleid zu reduzieren...)?

1 = Wesentlich weniger
2 = Weniger
3 = Neutral
4 = Mehr
5 = Wesentlich mehr

*

Bitte wählen Sie maximal eine Antwort.
Bitte wählen Sie die zutreffende Antwort für jeden Punkt aus:

1	2	3	4	5
○	○	○	○	○

Figure A1. Cont.

14. Was denken Sie, wie umweltfreundlich künstliches Fleisch gegenüber konventionellem Fleisch wäre (z. B. in der Lage, den ökologischen Fußabdruck der Landwirtschaft zu reduzieren wie: Wasserverbrauch, globale Erwärmung, Treibhausgasemissionen)?

1 = Wesentlich weniger
2 = Weniger
3 = Neutral
4 = Mehr
5 = Wesentlich mehr

*

Bitte wählen Sie maximal eine Antwort.
Bitte wählen Sie die zutreffende Antwort für jeden Punkt aus:

1	2	3	4	5
○	○	○	○	○

15. Glauben Sie, künstliches Fleisch könnte negative Auswirkungen auf die konventionelle Nutztierhaltung und die Fleischindustrie haben (z. B. Beschäftigungsverhältnisse...)?

1 = Wesentlich weniger / Stimme gar nicht zu
2 = Weniger / Stimme nicht zu
3 = Neutral / Stimme weder zu noch nicht zu
4 = Mehr / Stimme zu
5 = Wesentlich mehr / Stimme absolut zu

*

Bitte wählen Sie maximal eine Antwort.
Bitte wählen Sie die zutreffende Antwort für jeden Punkt aus:

1	2	3	4	5
○	○	○	○	○

16. Glauben Sie, künstliches Fleisch könnte negative Auswirkungen auf ländliche Regionen und ländliches Leben haben (z. B. Biodiversität, Tourismus, Landschaftspflege, Stabilität ländlicher Räume)?

1 = Wesentlich weniger / Stimme gar nicht zu
2 = Weniger / Stimme nicht zu
3 = Neutral / Stimme weder zu noch nicht zu
4 = Mehr / Stimme zu
5 = Wesentlich mehr / Stimme absolut zu

*

Bitte wählen Sie maximal eine Antwort.
Bitte wählen Sie die zutreffende Antwort für jeden Punkt aus:

1	2	3	4	5
○	○	○	○	○

Produkteigenschaften

17. Was denken Sie, wie gesund, sicher und nährstoffreich künstliches Fleisch gegenüber konventionellem Fleisch wäre (mit Hinblick auf Proteine, Vitamine...)?

1 = Wesentlich weniger
2 = Weniger
3 = Neutral
4 = Mehr
5 = Wesentlich mehr

*

Bitte wählen Sie maximal eine Antwort.
Bitte wählen Sie die zutreffende Antwort für jeden Punkt aus:

1	2	3	4	5
○	○	○	○	○

18. Was denken Sie, wie gut künstliches Fleisch gegenüber konventionellem Fleisch schmecken wird (z. B. ähnlich dem Geschmack von konventionellem Fleisch)?

1 = Wesentlich weniger
2 = Weniger
3 = Neutral
4 = Mehr
5 = Wesentlich mehr

*

Bitte wählen Sie maximal eine Antwort.
Bitte wählen Sie die zutreffende Antwort für jeden Punkt aus:

1	2	3	4	5
○	○	○	○	○

Figure A1. Cont.

Potentielle Interessen

19. Würden Sie künstliches Fleisch zukünftig als eine realisierbare Alternative zu konventionellem Fleisch, zu anderen Fleischersatzprodukten (wie Sojaproteine) oder anderen Lösungsansätzen (wie Reduzierung von Lebensmittelabfällen oder die Weiterentwicklung landwirtschaftlicher Produktionsmethoden) akzeptieren? *

❶ Bitte wählen Sie eine der folgenden Antworten:
Bitte wählen Sie nur eine der folgenden Antworten aus:

◯ Ja und ich esse bereits Fleischersatzprodukte / -alternativen
◯ Ja, aber ich esse keine Fleischersatzprodukte / -alternativen
◯ Nein, aber ich esse bereits Fleischersatzprodukte / -alternativen
◯ Nein und ich esse keine Fleischersatzprodukte / -alternativen

20. Was wären Gründe für Sie, um künstliches Fleisch zu probieren? *

❶ Bitte wählen Sie die zutreffenden Antworten aus:
Bitte wählen Sie alle zutreffenden Antworten aus:

☐ Als Lösung, um die wachsende Bevölkerung zu ernähren
☐ Attraktive Preise im Vergleich zu konventionellem Fleisch
☐ Ethische Gründe (Tierwohl erhöhen und Tierschlachtung verringern)
☐ Geringere Risiken von Zoonosen (Krankheiten, die vom Tier auf den Menschen übertragbar sind)
☐ Attraktivität neuer Technologien
☐ Sicheres Produkt
☐ Umweltfreundliches Produkt
☐ Neugier
☐ Ich bin nicht bereit, dieses Produkt zu probieren
☐ Sonstiges: ______

21. Warum würden Sie künstliches Fleisch nicht probieren wollen? *

❶ Bitte wählen Sie die zutreffenden Antworten aus:
Bitte wählen Sie alle zutreffenden Antworten aus:

☐ Unnatürlich
☐ Weniger schmackhaft / ansprechend
☐ Sorgen um Sicherheit
☐ Teurer als konventionelles Fleisch
☐ Gefühl von Abneigung (Ekel, Unbehagen)
☐ Negative Auswirkungen auf heimische Landwirte (Jobs…)
☐ Negative Auswirkungen auf ländliche Räume und ländliches Leben (Biodiversität, Tourismus, Landschaftspflege, Stabilität ländlicher Räume)
☐ Weniger Vertrauen in Labore und Start-ups im künstlichen Fleischsektor
☐ Erheblicher ökologischer Fußabdruck
☐ Sonstiges: ______

22. Was erwarten Sie von künstlichem Fleisch? *

❶ Bitte wählen Sie die zutreffenden Antworten aus:
Bitte wählen Sie alle zutreffenden Antworten aus:

☐ Adäquate Ernährung
☐ Geschmack ähnlich wie bei konventionellem Fleisch
☐ Sicherheit
☐ Günstiger als konventionelles Fleisch
☐ Geringerer ökologischer Fußabdruck
☐ Rückgang der Landwirtschaft
☐ Keine landwirtschaftliche Tätigkeit mehr
☐ Kein Tierleid
☐ Nichts
☐ Sonstiges: ______

Figure A1. Cont.

Wahrnehmung

23. Was halten Sie von künstlichem Fleisch? *

Bitte wählen Sie eine der folgenden Antworten:
Bitte wählen Sie nur eine der folgenden Antworten aus:

- Es ist vielversprechend und/oder akzeptabel
- Es ist spaßig und/oder faszinierend
- Es ist absurd und/oder ekelhaft

24. Verspüren Sie emotionalen Widerstand (Ekel, Angst) gegenüber dem Probieren von künstlichem Fleisch?

1 = Wesentlich weniger / Stimme gar nicht zu
2 = Weniger / Stimme nicht zu
3 = Neutral / Stimme weder zu noch nicht zu
4 = Mehr / Stimme zu
5 = Wesentlich mehr / Stimme absolut zu

*

Bitte wählen Sie maximal eine Antwort.
Bitte wählen Sie die zutreffende Antwort für jeden Punkt aus:

1	2	3	4	5
○	○	○	○	○

25. Würden Sie künstliches Fleisch probieren? *

Bitte wählen Sie eine der folgenden Antworten:
Bitte wählen Sie nur eine der folgenden Antworten aus:

- Definitiv ja
- Wahrscheinlich ja
- Unsicher
- Wahrscheinlich nicht
- Definitiv nicht

26. In welchem Rahmen wären Sie bereit, künstliches Fleisch regelmäßig zu essen? *

Bitte wählen Sie die zutreffenden Antworten aus:
Bitte wählen Sie alle zutreffenden Antworten aus:

- Im Restaurant
- Zu Hause
- In Fertigprodukten (Lasagne, Hamburger)
- Ich möchte künstliches Fleisch nicht regelmäßig essen
- Sonstiges:

27. Was wären Sie bereit für künstliches Fleisch auszugeben, im Vergleich zu konventionellem Fleisch? *

Bitte wählen Sie eine der folgenden Antworten:
Bitte wählen Sie nur eine der folgenden Antworten aus:

- Viel weniger als für konventionelles Fleisch, sogar nichts
- Weniger als für konventionelles Fleisch
- Den gleichen Preis wie für konventionelles Fleisch
- Mehr als für konventionelles Fleisch
- Viel mehr als für konventionelles Fleisch

28. Denken Sie, dass künstliches Fleisch sich durchsetzen wird? *

Bitte wählen Sie eine der folgenden Antworten:
Bitte wählen Sie nur eine der folgenden Antworten aus:

- Kurzfristig: 1 bis 5 Jahre
- Mittelfristig: 6 bis 15 Jahre
- Langfristig: über 16 Jahre
- Niemals

Figure A1. Cont.

Entwicklungsstrategien

29. Welche Bezeichnung bei der Beschreibung von künstlichem Fleisch trifft für Sie am besten zu (darunter sind auch die am häufigsten verwendeten Namen von Unternehmen aus der Branche)? *

❶ Bitte wählen Sie die zutreffenden Antworten aus:
Bitte wählen Sie alle zutreffenden Antworten aus:

- ☐ Künstliches Fleisch
- ☐ In-vitro-Fleisch
- ☐ Clean Meat
- ☐ Kultiviertes Fleisch
- ☐ Zellfleisch
- ☐ Laborfleisch
- ☐ Synthetisches Fleisch
- ☐ Tierfreies Fleisch
- ☐ Schlachtfreies Fleisch

30. Inwieweit sind private Forschungsmodelle (von Start-ups) Ihrer Meinung nach zur potentiellen Weiterentwicklung von künstlichem Fleisch relevant?

1 = Wesentlich weniger
2 = Weniger
3 = Neutral
4 = Mehr
5 = Wesentlich mehr

*

❶ Bitte wählen Sie maximal eine Antwort.
Bitte wählen Sie die zutreffende Antwort für jeden Punkt aus:

1	2	3	4	5
○	○	○	○	○

31. Was denken Sie, in welchem Umfang die öffentliche Forschung in die Weiterentwicklung dieser Biotechnologie investieren (Zeit und Finanzierung) sollte?

1 = Wesentlich weniger
2 = Weniger
3 = Neutral
4 = Mehr
5 = Wesentlich mehr

*

❶ Bitte wählen Sie maximal eine Antwort.
Bitte wählen Sie die zutreffende Antwort für jeden Punkt aus:

1	2	3	4	5
○	○	○	○	○

32. Falls dieses Produkt eines Tages vermarktet wird, denken Sie, es sollte „Fleisch" heißen? *

❶ Bitte wählen Sie eine der folgenden Antworten:
Bitte wählen Sie nur eine der folgenden Antworten aus:

- ○ Ja
- ○ Nein

33. Sind Sie damit einverstanden, dass die von Ihnen zur Verfügung gestellten Angaben gespeichert werden? *

❶ Bitte wählen Sie eine der folgenden Antworten:
Bitte wählen Sie nur eine der folgenden Antworten aus:

- ○ Ja
- ○ Nein

Sie haben die Umfrage abgeschlossen. Wir bedanken uns herzlich bei Ihnen, dass Sie sich die Zeit genommen haben, die Fragen zu künstlichem Fleisch zu beantworten!

Für Nutzer von SurveyCircle (www.surveycircle.com):
Der Survey Code lautet: F4QN-HGGF-LNVP-UFTW

25.01.2022 – 08:58

Übermittlung Ihres ausgefüllten Fragebogens:
Vielen Dank für die Beantwortung des Fragebogens.

Figure A1. Cont. English-language version.

Survey about artificial meat

Survey about Artificial Meat

* Required

Introduction and context of the survey

Artificial meat, also known as in vitro meat, cultured meat, lab meat, clean meat and also synthetic meat, is a novel food produced in laboratories using animal muscle stem cells, but does not come directly from a living animal and which proliferate in culture. The production of artificial meat is the subject of media enthusiasm to feed the growing human population. In order to solve the increasing concerns about environment (global warming) and ethic problems (animal welfare, animal suffering and slaughtering) but also the weaknesses of the conventional meat production (limited farming resources and ever increasing population), scientific research is devoted to introduce and develop on a large scale artificial meat as a new meat product in the future.

This study is conducted by French researchers from INRAE (Theix, 63), ISARA (Lyon, 69) and Bordeaux Sciences-Agro (Bordeaux, 33). The research institute WING (Science and Innovation for Sustainable Poultry Production) of the University of Veterinary Medicine Hannover supports the study with regard to the survey of German consumers. The purpose of this survey is to study the opinion of consumers about this biotechnology and to investigate their preferences and/or their aversions. That is why you are being asked to participate in this research project by taking an online survey, which should not take more than 15 minutes of your time. In accordance with the code of ethics of survey organizations, be assured that your personal data and the information you will provide will be kept confidential and not used except within average results for the statistical objectives of the survey. You should be 18 years old at least to complete this survey, but you do not need any specific skills nor academic degrees. Clicking on the next button to access the questionnaire implies your consent to participate. However, you can stop answering the survey anytime if you like. In this case, the information you have provided will not be saved. Only the display at the end of the questionnaire after page 8 implies the archiving of information. For any questions, you can write to 'futurecellmeat@gmail.com' or for Germany at anne-katrin.jacobs@tiho-hannover.de

Principles of artificial meat production

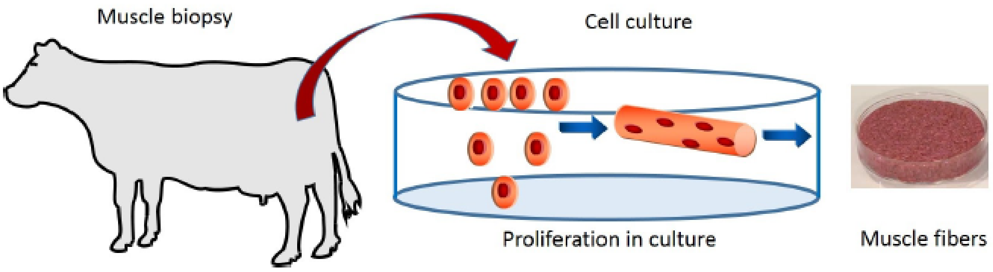

Demographic information

1. Sex *

Mark only one oval.

- ◯ Female
- ◯ Male
- ◯ Does not wish to answer

Figure A1. Cont. English-language version.

2. Continent or country of origin *

Mark only one oval.

- ◯ Germany
- ◯ Other countries within the European Union
- ◯ European countries outside the European Union
- ◯ China
- ◯ Russia
- ◯ Middle East and Asia excluding China and Asian Russia
- ◯ North America
- ◯ South America
- ◯ Africa
- ◯ Oceania

3. Age range *

Mark only one oval.

- ◯ 18-30 years of age
- ◯ 31-50 years of age
- ◯ 51 years of age and more

4. Education *

Mark only one oval.

- ◯ Secondary modern school
- ◯ University-Entrance diploma
- ◯ University degree
- ◯ PhD
- ◯ Don't want to answer
- ◯ Other: ____________________

Figure A1. Cont. English-language version.

5. Area of work *

Mark only one oval.

- ◯ Scientist within the meat sector
- ◯ Scientist outside the meat sector
- ◯ Not scientist but within the meat sector
- ◯ Not scientist and outside the meat sector

6. Monthly net income *

Mark only one oval.

- ◯ 1 500 € or less
- ◯ 1 500 - 2 000 €
- ◯ 2 000 - 2 500 €
- ◯ 2 500 - 3 000 €
- ◯ 3 000 - 4 000 €
- ◯ 4 000 € or more
- ◯ Does not wish to answer

7. Meat consumption *

Mark only one oval.

- ◯ Never: vegetarian or vegan diet
- ◯ Rarely: weekly or less
- ◯ Regularly: several times a week
- ◯ Daily or within each meal

Preamble

8. Have you ever heard about artificial meat before? *

Mark only one oval.

- ◯ Yes
- ◯ No

Figure A1. Cont. English-language version.

9. What are the most important criteria during your food shopping? *

Check all that apply.

- [ ] Ethics (breeding systems, production methods, animal welfare…)
- [ ] Environmental impacts
- [ ] Price
- [ ] Sensorial quality (taste, juiciness, tenderness…)
- [ ] Aspect
- [ ] Energy intake (calories)
- [ ] Nutritional value (proteins, vitamins…)
- [ ] Origin and traceability
- [ ] Safety
- [ ] Marks and/or labels

Other: [ ] ____________________

Societal challenges

10. Do you think the on-farm breeding and meat industry cause important ethical problems (e.g. animal suffering, slaughtering...) ? *

Mark only one oval.

	1	2	3	4	5	
Much less / I disagree	⬭	⬭	⬭	⬭	⬭	Much more / I agree

11. Do you think that the on-farm breeding and meat industry cause importantenvironmental problems (e.g.huge water consumption, greenhouse gas emissions...) ? *

Mark only one oval.

	1	2	3	4	5	
Much less / I disagree	⬭	⬭	⬭	⬭	⬭	Much more / I agree

Figure A1. Cont. English-language version.

12. To deal with these potential problems, do you think that reducing our meat consumption could be a good solution? *

Mark only one oval.

	1	2	3	4	5	
Much less / I disagree	◯	◯	◯	◯	◯	Much more / I agree

13. How ethical do you think artificial meat would be compared to conventional meat (e.g. able to significantly improve animal welfare, reduce animal suffering…)? *

Mark only one oval.

	1	2	3	4	5	
Much less / I disagree	◯	◯	◯	◯	◯	Much more / I agree

14. How eco-friendly do you think artificial meat would be compared to conventional meat (e.g. able to significantly reduce the environmental footprint of farming such as water consumption, global warming effect, greenhouse gas emissions…)? *

Mark only one oval.

	1	2	3	4	5	
Much less / I disagree	◯	◯	◯	◯	◯	Much more / I agree

15. Do you think artificial meat would have negative impacts on traditional livestock farming and meat industry (e.g. employment…)? *

Mark only one oval.

	1	2	3	4	5	
Much less / I disagree	◯	◯	◯	◯	◯	Much more / I agree

Figure A1. Cont. English-language version.

16. Do you think artificial meat would have negative impacts on territories and rural life (e.g. biodiversity, tourism, landscape maintenance, vitality of the territories…)? *

Mark only one oval.

	1	2	3	4	5	
Much less / I disagree	⬭	⬭	⬭	⬭	⬭	Much more / I agree

Characteristics of the product

17. How healthy, safe and with a high-nutritional-value do you think artificial meat would be compared to conventional meat (i.e. in terms of proteins, vitamins…)? *

Mark only one oval.

	1	2	3	4	5	
Much less	⬭	⬭	⬭	⬭	⬭	Much more

18. How tasty do you think artificial meat would be compared to conventional meat (i.e. similar to conventional meat in terms of taste)? *

Mark only one oval.

	1	2	3	4	5	
Much less	⬭	⬭	⬭	⬭	⬭	Much more

Potential interests

19. Would you accept artificial meat as a viable alternative compared to conventional meatin the future, compared toother meat substitutes (such as soy proteins) or othesolutions (such as reducing food waste or developing our farming practices)? *

Mark only one oval.

- ⬭ Yes and I already eat meat substitutes/alternatives
- ⬭ Yes but I don't eat meat substitutes/alternatives
- ⬭ No but I eat meat substitutes/alternatives
- ⬭ No and I don't eat meat substitutes/alternatives

Figure A1. Cont. English-language version.

20. What could be the reasons for you to try artificial meat? (multiple choice) *

Check all that apply.

- [ ] Solution to feed the ever-growing human population
- [ ] Attractive price compared to conventional meat
- [ ] Ethic (improve animal welfare and reduce animal slaughtering)
- [ ] Less risk of zoonosis (disease that can be transmitted from animals to people)
- [ ] Attractivity of high-tech technologies
- [ ] Clean product
- [ ] Eco-friendly product
- [ ] Curiosity
- [ ] I am not willing to try this product

Other: [ ] ______________________

21. Why would you NOT be willing to try artificial meat? (multiple choice) *

Check all that apply.

- [ ] Unnatural
- [ ] Less tasty/appealing
- [ ] Worries about safety
- [ ] More expensive than conventional meat
- [ ] Feeling of reluctance (disgust, nervousness ...)
- [ ] Negative impact on local farmers (jobs ...)
- [ ] Negative impact on territories and rural life (biodiversity, tourism, landscape maintenance, vitality of territories ...)
- [ ] Less trust in laboratories and artificial meat start-ups
- [ ] Significant environmental footprints

Other: [ ] ______________________

22. What would you expect from artificial meat? (multiple choice) *

Check all that apply.

- [ ] Adequate nutrition
- [ ] Tasty/Taste similar to conventional meat
- [ ] Safety
- [ ] Less expensive than conventional meat
- [ ] Less environmental footprints
- [ ] Reduction of farming
- [ ] No farming
- [ ] No animal pain/suffering
- [ ] Nothing

Other: [ ] ______________________

Figure A1. Cont. English-language version.

Perception

23. What do you think of artificial meat? *

Mark only one oval.

- ◯ It is promising and/or acceptable
- ◯ It is fun and/or intriguing
- ◯ It is absurd and/or disgusting

24. Do you have emotional resistance (e.g. disgust, nervousness) to try artificial meat? *

Mark only one oval.

	1	2	3	4	5	
Much less	◯	◯	◯	◯	◯	Much more

25. Would you be willing to try artificial meat? *

Mark only one oval.

- ◯ Definitely yes
- ◯ Probably yes
- ◯ Unsure
- ◯ Probably not
- ◯ Definitely not

26. In which context(s), would you be willing to eat artificial meat regularly? (multiple choice) *

Check all that apply.

- ☐ At the restaurant
- ☐ At home
- ☐ In ready-to-eat meals: lasagna, burger…
- ☐ I do not want to eat artificial meat regularly

Other: ☐ ____________________

Figure A1. Cont. English-language version.

27. How much would you be willing to pay for artificial meat compared to conventional meat? *

Mark only one oval.

- Much less than conventional meat, even nothing at all
- Less than conventional meat
- Same price as conventional meat
- More than conventional meat
- Much more than conventional meat

28. Do you think artificial meat is realistic? *

Mark only one oval.

- On the short term: from 1 to 5 years
- On the medium term: from 6 to 15 years
- On the long term: more than 16 years
- Never

Development strategies

29. Which names are the most relevant to you to qualify artificial meat? (among the names most commonly used by companies in the sector) (multiple choice) *

Check all that apply.

- Artificial meat
- In vitro meat
- Clean meat
- Cultured meat
- Cellular meat
- Lab meat
- Synthetic meat
- Animal-free meat
- Slaughter-free meat

30. To which extent do you think that the private research model (by start-ups) is relevanfor potentially developing research on artificial meat? *

Mark only one oval.

	1	2	3	4	5	
Much less						Much more

Figure A1. Cont. English-language version.

31. To which extent do you think that scientific public research must invest (time and funding) to develop this biotechnology? *

Mark only one oval.

	1	2	3	4	5	
Much less	◯	◯	◯	◯	◯	Much more

32. If this product is commercialized one day, do you think it should be named "meat"?

Mark only one oval.

◯ Yes
◯ No

33. Do you agree that the information you provided will be saved

Mark only one oval.

◯ Yes
◯ No

Supplementary Table A1. Responses about societal challenges related to the meat industry and perceptions about artificial meat (*n*=3,558).

**A. Questions-Societal Challenges**	**Responses (1 = Much less, 2 = Less, 3 = Neutral, 4 = More, 5 = Much more)**											
	1		2		3		4		5		Mean	SD
	No.	%	No.	%	No.	%	No.	%	No.	%		
Does the conventional meat industry have ethical problems?	207	5.8	266	7.5	281	7.9	732	20.6	2072	58.2	4.18	1.21
Does the conventional meat industry have environmental problems?	204	5.7	251	7.1	263	7.4	718	20.2	2122	59.6	4.21	1.19
Could reducing meat consumption be a good solution to resolve above problems?	269	7.6	244	6.9	243	6.8	739	20.8	2063	58.0	4.15	1.25
How ethical artificial meat would be compared to conventional meat?	359	10.1	268	7.5	560	15.7	1052	29.6	1319	37.1	3.76	1.29
How eco-friendly artificial meat would be compared to conventional meat?	303	8.5	334	9.4	869	24.4	1319	37.1	733	20.6	3.52	1.16
Does artificial meat have negative impacts on conventional meat industry?	263	7.4	518	14.6	849	23.9	1307	36.7	621	17.5	3.42	1.15
Does artificial meat have negative impacts on territories and rural life?	765	21.5	972	27.3	643	18.1	671	18.9	507	14.2	2.77	1.35

**B. Questions-Perceptions**	**Responses (1 = Much less, 2 = Less, 3 = Neutral, 4 = More, 5 = Much more)**											
	1		2		3		4		5		Mean	SD
	No.	%	No.	%	No.	%	No.	%	No.	%		
How healthy, safe and nutritional artificial meat would be compared to conventional meat?	401	11.3	516	14.5	1718	48.3	631	17.7	292	8.2	2.97	1.04
How tasty artificial meat would be compared to conventional meat?	400	11.2	744	20.9	1873	52.6	363	10.2	178	5.0	2.77	0.95
Do you have emotional resistance to try artificial meat?	1266	35.6	624	17.5	617	17.3	529	14.9	522	14.7	2.56	1.46

Supplementary Table A2. Pairwise comparisons between significant demographic groups for willingness to try (WTT) artificial meat (on a scale from 5 to 1).

**Sex**		**Age**					**Income**							**Meat consumption**		
	18-30	31-50	≥ 51		< €1,500	€1,500-€2,000	€2,000-€2,500	€2,500-€3,000	> €3,000-€4,000	> €4,000	Does not wish to answer		Never	Rarely	Regularly	Daily
Female	3.89^{d}	3.76^{bc}	3.57^{a}		3.74^{d}	3.91^{abcd}	3.84^{cd}	3.82^{abc}	3.94^{abc}	3.77^{abc}	3.57^{ab}		3.48^{bc}	4.17^{d}	3.83^{a}	3.20^{a}
Male	4.10^{cd}	3.73^{bc}	3.31^{ab}		4.20^{c}	3.71^{cd}	3.84^{cd}	3.77^{cd}	3.57^{cd}	3.55^{bcd}	3.21^{abc}		3.84^{a}	4.41^{d}	3.50^{c}	3.31^{ab}
Don't want to answer	4.05^{abcd}	3.16^{abc}	2.67^{ab}		3.93^{abcd}	4.43^{abcd}	3.86^{abcd}	3.00^{abcd}	3.10^{abcd}	3.00^{abcd}	2.53^{c}		3.75^{abcd}	3.57^{abcd}	2.89^{a}	3.00^{abcd}

**Age**		**Education**							**Meat consumption**		
	Secondary modern school	University-entrance diploma	University degree	PhD	Don't want to answer	Others		Never	Rarely	Regularly	Daily
18-30	3.16^{ab}	3.85^{cd}	4.10^{d}	4.44^{cd}	3.00^{abcd}	4.1^{bcd}		3.62^{bc}	4.40^{a}	4.08^{d}	3.30^{abc}
31-50	3.21^{ab}	3.61^{bc}	3.86^{cd}	4.08^{d}	2.75^{ab}	3.46^{abcd}		3.57^{bc}	4.20^{de}	4.20^{bc}	3.27^{ab}
≥ 51	3.46^{b}	3.20^{ab}	3.38^{b}	3.95^{cd}	2.06^{a}	2.67^{abcd}		3.44^{abc}	3.92^{cd}	3.23^{a}	3.26^{abc}

**Education**			**Income**				
	< €1,500	€1,500-€2,000	€2,000-€2,500	€2,500-€3,000	€3,000-€4,000	> €4,000	Does not wish to answer
Secondary modern school	3.34^{abcde}	3.39^{abcdef}	3.44^{abcdefghij}	3.39^{abcdefghij}	3.33^{abcdefghij}	2.52^{abc}	2.74^{ab}
University-entrance diploma	3.82^{efghij}	3.87^{efghij}	3.75^{defghij}	3.44^{abcdefghij}	2.96^{abcd}	3.26^{abcdefgh}	3.59^{bcdefgh}
University degree	4.03^{hj}	4.03^{fghij}	3.94^{efghij}	3.90^{efghij}	3.86^{efghij}	3.59^{efghij}	3.52^{abcdefgi}
PhD	4.00^{bcdefghij}	4.00^{bcdefghij}	4.44^{ij}	4.00^{efghij}	4.08^{efghij}	4.15^{ghij}	3.73^{bcdefghij}
Don't want to answer	2.5^{abcdefghij}	2.25^{abcdefghij}	3.25^{abcdefghij}	5.00^{abcdefghij}	3.33^{abcdefghij}	2.14^{abcdefgh}	2.26^{a}
Others	3.9^{abcdefghij}	4.67^{abcdefghij}	3.00^{abcdefghij}	4.50^{abcdefghij}	3.50^{abcdefghij}	4.00^{abcdefghij}	2.67^{abcdefghij}

Table A2. Cont.

Area of work		Income					
	< €1,500	€1,500-€2,000	€2,000-€2,500	€2,500-€3,000	€3,000-€4,000	> €4,000	Does not wish to answer
Scientist: meat sector	$4.06^{abcdefg}$	$4.33^{abcdefg}$	4.45^{bcdefg}	$4.17^{abcdefg}$	$3.47^{abcdefg}$	4.32^{cdefg}	$4.36^{abcdefg}$
Scientist: other sector	4.32^{g}	4.27^{fg}	4.07^{efg}	3.82^{bcdefg}	3.73^{bcdefg}	3.75^{bcdefg}	$3.64^{abcdefg}$
Worker: meat sector	3.05^{abcd}	2.81^{ab}	$3.57^{abcdefg}$	$3.70^{abcdefg}$	$3.70^{abcdefg}$	3.45^{abcdef}	2.73^{a}
Worker: other sector	3.82^{def}	3.83^{defg}	3.80^{cdef}	3.78^{cdef}	3.77^{bcdef}	3.56^{bcde}	3.41^{abc}

Income		Meat consumption		
	Never	Rarely	Regularly	Daily
< €1,500	3.48^{abcd}	4.32^{j}	3.99^{efghij}	$3.76^{abcdefghij}$
€1,500 - €2,000	$3.69^{abcdefg}$	4.30^{hij}	$3.71^{abcdefgh}$	$3.52^{abcdefghij}$
€2,000 - €2,500	3.61^{abcde}	4.34^{ij}	$3.81^{cdefghi}$	3.29^{abcdef}
€2,500 - €3,000	$3.78^{abcdefghij}$	4.26^{fghij}	3.69^{abcdef}	2.74^{a}
€3,000 - €4,000	$3.92^{cdefghij}$	$4.01^{defghij}$	3.51^{abcde}	$3.58^{abcdefghij}$
> €4,000	$3.55^{abcdefghi}$	4.34^{ghij}	3.43^{abcd}	3.07^{abc}
Does not wish to answer	3.17^{ab}	$3.87^{bcdefghij}$	3.32^{abc}	2.92^{abcd}

AM: artificial meat
Willingness to try AM: 5= Definitely yes; 4= Probably yes; 3= Uncertain; 2= Probably not; 1= Definitely not.
Groups with different code letters (a-j) differ significantly at the 0.05 level; to correct for multiple testing, a Bonferroni adjustment was used.

Supplementary Table A3. Pairwise comparisons between significant demographic groups for willingness to eat artificial meat regularly (WTE) (on a scale from 0 to 1).

Sex		Age					Income							Meat consumption		
	18-30	31-50	≥ 51		< €1,500	€1,500-€2,000	€2,000-€2,500	€2,500-€3,000	€3,000-€4,000	> €4,000	Does not wish to answer		Never	Rarely	Regularly	Daily
Female	0.59^{a}	0.54^{b}	0.41^{c}		0.55^{a}	0.56^{ab}	0.57^{ab}	0.55^{bc}	0.57^{bc}	0.48^{bc}	0.45^{bc}		0.45^{bcd}	0.65^{a}	0.55^{de}	0.47^{cde}
Male	0.69^{b}	0.53^{b}	0.34^{c}		0.70^{b}	0.58^{ab}	0.55^{ab}	0.50^{ab}	0.46^{ab}	0.43^{bc}	0.43^{bc}		0.56^{e}	0.72^{ab}	0.46^{c}	0.43^{bcde}
Don´t want to answer	0.50^{abc}	0.54^{bc}	0.20^{bc}		0.57^{abc}	0.57^{abc}	0.57^{abc}	0.25^{abc}	0.30^{abc}	0.20^{abc}	0.11^{c}		0.50^{abcde}	0.44^{abcde}	0.19^{e}	0.25^{abcde}

Age			Income							Meat consumption		
	< €1,500	€1,500-€2,000	€2,000-€2,500	€2,500-€3,000	€3,000-€4,000	> €4,000	Does not wish to answer		Never	Rarely	Regularly	Daily
18-30	0.64^{a}	0.61^{ab}	0.60^{abc}	0.63^{abcd}	0.58^{abcdef}	0.44^{abcdef}	0.58^{abcd}		0.50^{d}	0.76^{a}	0.66^{ab}	0.49^{bcde}
31-50	0.55^{abcde}	0.58^{abcd}	0.58^{abc}	0.55^{abcd}	0.55^{abcd}	0.45^{bcdef}	0.40^{def}		0.49^{d}	0.64^{abc}	0.51^{d}	0.45^{de}
≥ 51	0.26^{f}	0.35^{cdef}	0.44^{bcdef}	0.33^{ef}	0.39^{def}	0.44^{bcdef}	0.33^{f}		0.36^{de}	0.50^{cd}	0.32^{e}	0.35^{de}

Income		Meat consumption		
	Never	Rarely	Regularly	Daily
< €1,500	0.46^{defg}	0.74^{a}	0.62^{abc}	0.68^{abcde}
€1,500-€2,000	0.53^{bcdefg}	0.67^{abc}	0.50^{bcdefg}	0.66^{abcdefg}
€2,000-€2.500	0.50^{bcdefg}	0.71^{ab}	0.56^{bcdef}	0.38^{bcdefg}
€2.500-€3,000	0.50^{bcdefg}	0.64^{abcd}	0.52^{bcdefg}	0.19^{fg}
€3,000-€4,000	0.53^{abcdefg}	0.54^{abcdefg}	0.49^{cdefg}	0.44^{abcdefg}
> €4,000	0.40^{cdefg}	0.69^{abc}	0.35^{g}	0.36^{cdefg}
Does not wish to answer	0.37^{efg}	0.54^{abcdefg}	0.42^{efg}	0.28^{cdefg}

AM: artificial meat
Willingness to eat AM: 0= Not willing to eat AM regularly; 1= Willing to eat AM regularly.
Groups with different code letters (a-g) differ significantly at the 0.05 level; to correct for multiple testing, a Bonferroni adjustment was used.

Supplementary Table A4. Pairwise comparisons between significant demographic groups for willingness to pay for artificial meat (WTP) (on a scale from 1 to 5).

**Gender**		**Age**					**Income**							**Meat consumption**		
	18-30	31-50	≥ 51		< €1,500	€1,500-€2,000	€2,000-€2,500	€2,500-€3,000	€3,000-€4,000	> €4,000	Does not wish to answer		Never	Rarely	Regularly	Daily
Female	3.09^{ab}	2.92^{c}	2.69^{d}		3.06^{ab}	2.97^{bcdefg}	2.94^{abcd}	2.88^{cdefgh}	2.98^{defgh}	2.80^{egh}	2.77^{egh}		3.41^{a}	2.92^{b}	2.53^{d}	2.08^{de}
Male	2.94^{a}	2.65^{b}	2.29^{c}		3.01^{a}	2.69^{abc}	2.79^{abc}	2.64^{abcd}	2.57^{abc}	2.39^{abcd}	2.28^{bcdf}		3.52^{a}	3.09^{b}	2.23^{c}	2.23^{de}
Don´t want to answer	3.20^{abc}	2.03^{d}	1.60^{d}		$3.07^{abcdefgh}$	3.57^{abcde}	$2.57^{abcdefgh}$	$1.75^{abcdefgh}$	1.60^{fgh}	$1.60^{abcdefgh}$	1.79^{h}		3.30^{ab}	2.38^{bcde}	1.54^{e}	1.75^{bcde}

**Age**		**Education**								**Income**				
	Secondary modern school	University-entrance diploma	University degree	PhD	Don´t want to answer	Others		< €1,500	€1,500-€2,000	€2,000-€2,500	€2,500-€3,000	€3,000-€4,000	> €4,000	Does not wish to answer
18-30	2.51^{cdef}	3.11^{a}	3.06^{a}	3.07^{abcd}	2.00^{abcdef}	3.00^{abcdef}		3.15^{a}	3.00^{ab}	2.99^{ab}	2.99^{abcd}	$2.77^{abcdefg}$	$2.33^{abcdefg}$	$2.84^{abcdefg}$
31-50	2.55^{def}	2.71^{cde}	2.85^{bc}	3.03^{ab}	1.95^{ef}	2.55^{abcdef}		2.91^{abc}	2.84^{bcdef}	2.92^{ab}	2.81^{bcdef}	2.87^{bcde}	2.55^{cdefg}	2.51^{defg}
≥ 51	2.43^{ef}	2.30^{f}	2.53^{def}	2.64^{cdef}	2.11^{cdef}	1.78^{cdef}		2.38^{efg}	$2.68^{abcdefg}$	2.53^{bcdefg}	2.38^{fg}	2.48^{defg}	2.52^{defg}	2.39^{g}

**Age**		**Meat consumption**		
	Never	Rarely	Regularly	Daily
18-30	3.46^{a}	3.06^{b}	2.63^{c}	2.22^{de}
31-50	3.43^{a}	2.92^{b}	2.38^{d}	2.02^{e}
≥ 51	3.37^{a}	2.80^{bc}	2.08^{e}	2.04^{de}

Table A4. Cont.

**Education**			**Income**				
	< €1,500	€1,500-€2,000	€2,000-€2,500	€2,500-€3,000	€3,000-€4,000	> €4,000	Does not wish to answer
Secondary modern school	2.59^{bcdef}	2.62^{bcdef}	2.57^{bcdef}	2.50^{bcdef}	2.24^{ef}	2.04^{f}	2.45^{bcdef}
University-entrance diploma	3.09^{a}	2.90^{abcdef}	2.94^{abcde}	2.41^{cdef}	2.26^{f}	2.33^{cdef}	2.68^{abcdef}
University degree	3.09^{a}	3.02^{ab}	2.94^{abc}	2.83^{abcdef}	2.85^{abcdef}	2.51^{def}	2.60^{cdef}
PhD	3.00^{abcdef}	2.91^{abcdef}	3.12^{abcd}	2.99^{abcde}	3.04^{abc}	2.79^{abcdef}	2.58^{abcdef}
Don´t want to answer	2.00^{abcdef}	1.75^{abcdef}	2.00^{abcdef}	3.00^{abcdef}	2.00^{abcdef}	1.86^{abcdef}	2.11^{bcdef}
Others	3.57^{abcdef}	2.67^{abcdef}	2.00^{abcdef}	3.00^{abcdef}	2.17^{abcdef}	1.00^{abcdef}	2.00^{abcdef}

**Income**		**Meat consumption**		
	Never	Rarely	Regularly	Daily
< €1,500	3.41^{ab}	3.05^{defg}	2.58^{jklm}	2.32^{jklmn}
€1,500-€2,000	3.43^{abc}	2.99^{afghi}	2.41^{klmn}	2.34^{hijklmn}
€2,000-€2,500	3.51^{a}	3.04^{cdefgh}	2.39^{klmn}	1.97^{mn}
€2,500-€3,000	3.45^{abcd}	2.93^{efghij}	2.41^{klmn}	1.74^{n}
€3,000-€4,000	3.53^{ab}	2.79^{fghijk}	2.35^{lmn}	2.27^{ijklmn}
> €4,000	3.42^{abcde}	3.03^{bcdefghi}	2.15^{n}	1.98^{n}
Does not wish to answer	3.29^{abcdef}	2.68^{ghijkl}	2.21^{n}	2.00^{lmn}

AM: artificial meat

Willingness to pay for AM: 1= Much less; 2= Less; 3= Same; 4= More; 5= Much more

Groups with different code letters (a-n) differ significantly at the 0.05 level; to correct for multiple testing, a Bonferroni adjustment was used.

Supplementary Table A5. Terms chosen by respondents to describe artificial meat (% rounded, multiple choice).

**Designation**	**%**
Artificial meat	30
In vitro meat	28
Clean meat	25
Laboratory meat	24
Synthetic meat	22
Slaughter free meat	20
Animal free meat	19
Cultured meat	19
Cell meat	10

Supplementary Table A6. Most important criteria when purchasing food (multiple choice).

**Answers**	**n**	**%**
Ethics	2,637	74.1
Sensorial quality	2,198	61.8
Origin and back tracking	2,021	56.8
Environmental impact	1,851	52.0
Price	1,593	44.8
Appearance	1,308	36.8
Nutritional value	1,202	33.8
Safety	941	26.4
Calory	478	13.4
Brands	227	6.4
